# Factors predicting the transition from acute to persistent pain in people with ‘sciatica’: the FORECAST longitudinal prognostic factor cohort study protocol

**DOI:** 10.1136/bmjopen-2023-072832

**Published:** 2023-04-05

**Authors:** Annina B Schmid, Lucy Ridgway, Louise Hailey, Mohamed Tachrount, Fay Probert, Kathryn R Martin, Whitney Scott, Geert Crombez, Christine Price, Claire Robinson, Soraya Koushesh, Sarim Ather, Brigitte Tampin, Marco Barbero, Daniel Nanz, Stuart Clare, Jeremy Fairbank, Georgios Baskozos

**Affiliations:** 1 Nuffield Department of Clinical Neurosciences, University of Oxford, Oxford, Oxfordshire, UK; 2 Wellcome Centre for Integrative Neuroimaging, University of Oxford, Oxford, Oxfordshire, UK; 3 Nuffield Department of Orthopaedics Rheumatology and Musculoskeletal Sciences, University of Oxford, Oxford, Oxfordshire, UK; 4 Department of Chemistry, University of Oxford, Oxford, Oxfordshire, UK; 5 Academic Primary Care, Institute of Applied Health Sciences, School of Medicine, Medical Sciences and Nutrition, University of Aberdeen, Aberdeen, UK; 6 Aberdeen Centre for Arhtritis and Musculoskeletal Health, School of Medicine, Medical Sciences and Nutrition, University of Aberdeen, Aberdeen, UK; 7 Health Psychology Section, Institute of Psychiatry, Psychology and Neuroscience, King's College London, London, UK; 8 INPUT Pain Management Unit, Guy's and St Thomas' NHS Foundation Trust, London, UK; 9 Department of Experimental Clinical and Health Psychology, University of Ghent, Gent, Belgium; 10 Patient partner FORECAST study, Oxford University, Oxford, UK; 11 Department of Radiology, Oxford University Hospitals NHS Foundation Trust, Oxford, Oxfordshire, UK; 12 Department of Physiotherapy, Sir Charles Gairdner Hospital, Perth, Western Australia, Australia; 13 Curtin School of Allied Health, Faculty of Health Sciences, Curtin University, Perth, Western Australia, Australia; 14 Faculty of Business and Social Sciences, University of Applied Sciences, Osnabrueck, Germany; 15 Rehabilitation Research Laboratory 2rLab, Department of Business Economics Health and Social Care, University of Applied Sciences and Arts of Southern Switzerland, Manno, Ticino, Switzerland; 16 Swiss Center for Musculoskeletal Imaging, Balgrist Campus AG, Zurich, Zurich, Switzerland; 17 Medical Faculty, University of Zurich, Zurich, Zurich, Switzerland

**Keywords:** Chronic Pain, Neurology, Rehabilitation medicine, Rheumatology, Neurological injury, Neurological pain

## Abstract

**Introduction:**

Sciatica is a common condition and is associated with higher levels of pain, disability, poorer quality of life, and increased use of health resources compared with low back pain alone. Although many patients recover, a third develop persistent sciatica symptoms. It remains unclear, why some patients develop persistent sciatica as none of the traditionally considered clinical parameters (eg, symptom severity, routine MRI) are consistent prognostic factors.

The FORECAST study (factors predicting the transition from acute to persistent pain in people with ‘sciatica’) will take a different approach by exploring mechanism-based subgroups in patients with sciatica and investigate whether a mechanism-based approach can identify factors that predict pain persistence in patients with sciatica.

**Methods and analysis:**

We will perform a prospective longitudinal cohort study including 180 people with acute/subacute sciatica. N=168 healthy participants will provide normative data. A detailed set of variables will be assessed within 3 months after sciatica onset. This will include self-reported sensory and psychosocial profiles, quantitative sensory testing, blood inflammatory markers and advanced neuroimaging. We will determine outcome with the Sciatica Bothersomeness Index and a Numerical Pain Rating Scale for leg pain severity at 3 and 12 months.

We will use principal component analysis followed by clustering methods to identify subgroups. Univariate associations and machine learning methods optimised for high dimensional small data sets will be used to identify the most powerful predictors and model selection/accuracy.

The results will provide crucial information about the pathophysiological drivers of sciatica symptoms and may identify prognostic factors of pain persistence.

**Ethics and dissemination:**

The FORECAST study has received ethical approval (South Central Oxford C, 18/SC/0263). The dissemination strategy will be guided by our patient and public engagement activities and will include peer-reviewed publications, conference presentations, social media and podcasts.

**Trial registration number:**

ISRCTN18170726; Pre-results.

**STRENGTHS AND LIMITATIONS OF THIS STUDY**

- This study has the potential to advance our understanding of the heterogeneity of pathomechanisms in people with sciatica and to identify factors that predict pain persistence.
- This data set will include the largest deeply phenotyped ‘sciatica’ cohort to date.
- Harmonisation with the PAINSTORM consortium (Partnership for Assessment and Investigation of Neuropathic Pain: Studies Tracking Outcomes, Risks and Mechanisms) will afford integration of the FORECAST cohort (factors predicting the transition from acute to persistent pain in people with ‘sciatica’) into a much larger dataset of neuropathic pain.
- The large amount of data points collected for a modest cohort size will pose challenges for analyses and will require dimensionality reduction techniques.
- Patient recruitment will be challenging given the time intensive phenotyping protocol. This may lead to recruitment bias.

## Introduction

Low back pain (LBP) is associated with more disability than any other condition.^1^ Up to 60% of patients with LBP also experience leg pain, which is associated with worse health outcomes. In some cases, the leg pain is caused by nerve root involvement, commonly referred to as ‘sciatica,’ whereas some patients with ‘sciatica’ have pain of predominantly nociceptive character, others develop neuropathic (nerve related) pain, which is characterised by burning pain, electric shocks or tingling. The presence of neuropathic pain in sciatica further increases suffering and disability.^2^ The management of sciatica is, therefore, a priority. The National Institute for Health and Care Excellence (NICE) guidelines recommend a period of non-invasive treatment (eg, medication, physiotherapy) before invasive treatment (eg, surgery) is considered.^3^ Sadly, first-line management for patients with sciatica remains largely ineffective^4 5^ and at least one-third develops persistent pain and disability lasting a year or longer.^6–10^

It remains unclear why some patients develop persistent sciatica. Two recent systematic reviews have established that none of the traditionally considered clinical parameters (eg, pain intensity, routine MRI, mental well-being) are consistent prognostic factors.^11 12^ Since those publications, the largest prognostic study in patients with sciatica in primary care^8^ identified several factors that are weakly associated with improvement, these included shorter pain duration, belief that symptoms will not last long, myotomal weakness, overall impact of sciatica. However, at 12 months, only two factors were independently associated with outcome in the multivariable model analysis. This restricts the usefulness of predictive modelling for risk estimation of outcome for individual patients. The absence of prognostic factors hinders the early identification of patients at risk of developing persistent pain and prevents personalised treatments.

These challenges in management and risk prediction are partly attributed to a lack of understanding of the pathomechanisms at play in sciatica. Sciatica is a heterogeneous condition likely caused by differing mechanisms in individual patients,^13^ which are potentially amenable to targeted treatment. In the field of neuropathic pain, mechanism-based stratification using deep phenotyping has been advocated to facilitate personalised pain management.^14^ In contrast to traditionally used methods that quantify the severity of the disease with a limited battery of basic clinical measures (eg, routine MRI scans, symptom severity basic questionnaires), a mechanism-based approach aims to stratify patients by the distinct underlying mechanisms. It has been suggested that the nature of the pathomechanisms at play in patients with pain may influence treatment outcome and prognosis.^14–16^ The utility of such a mechanism-based approach in predicting pain persistence in people with sciatica remains unknown.

The FORECAST study will examine the value of a mechanism-based deep phenotyping approach including main domains assessing nerve function, nerve structure, inflammation and psychosocial factors.

The aims of the FORECAST study are:

1. To explore mechanism-based subgroups in patients with acute/subacute sciatica.
2. To investigate whether a mechanism-based approach can identify factors that predict pain persistence in people with sciatica.

## Methods

The FORECAST study is a prospective longitudinal prognostic factor cohort study that is based on feasibility data and closely informed by patient and public involvement and engagement (PPIE) activities including feedback from our named patient partners, six-member patient advisory group (PAG) and survey results from participants of the feasibility study. The study will be performed and reported according to the guidance for strenghtening the reporting of observational studies in epidemiology (STROBE)^17^ and the statement for transparent reporting of a multivariable prediction model for individual prognosis or diagnosis (TRIPOD).^18^

### Participants

We will include n=180 patients with acute/subacute ‘sciatica’ and n=168 healthy age and gender-matched participants without symptoms of sciatica/LBP. Healthy participants are important to establish normative values for blood markers, somatosensory profiling and neuroimaging.

People aged >18 years with a clinical diagnosis of ‘sciatica’ will be recruited from primary care in Oxfordshire (eg, primary care providers for the National Health Service as wellGeneral Practitioners, Physiotherapy, Osteopathy and Chiropractor clinics) and through leaflets on public noticeboards. Sciatica symptom onset of the current episode needs to be within the past 3 months with a symptom-free period of at least 3 months preceding the current sciatica symptoms. The inclusion criteria for patients with ‘sciatica’ are based on a published diagnostic model,^19^ which includes five weighted parameters (self-reported sensory changes, below knee pain, leg pain worse than back pain, neurodynamic tests and neurological deficit). A sum score >4 will be defined as sciatica, with a mean predicted probability of 83%. In addition, patients with suspected sciatica will undergo a clinical examination by a physiotherapist to further confirm the diagnosis of sciatica and rule out other diagnoses (see the section additional phenotypic data below).

The following exclusion criteria will apply; presence of other nerve-related disorders (eg, diabetic neuropathy, stroke), previous lumbar spine surgery, serious spinal diseases (eg, infection, cauda equina syndrome, metastatic lesions), chronic inflammatory disorders, other pain conditions that may confound assessment (eg, fibromyalgia), pregnancy, insufficient command of the English language to obtain consent/complete questionnaires and contraindications to MRI for those selected for scanning.

### Study procedure

**Figure 1:**
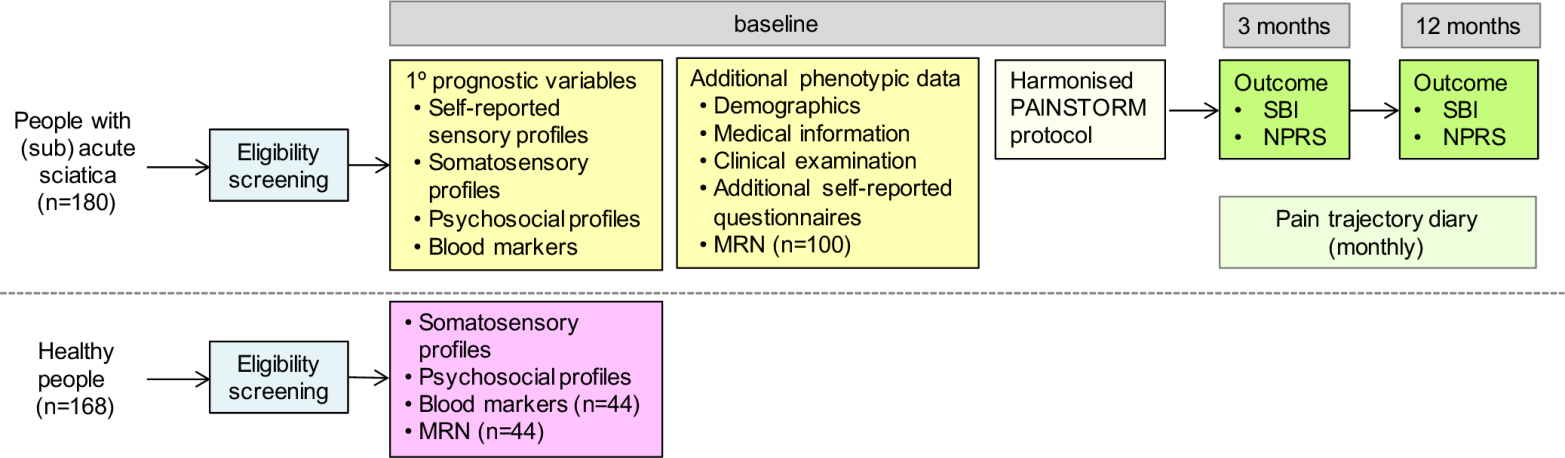
Study flow diagram. MRN, magnetic resonance neurography; NPRS, Numerical Pain Rating Scale; SBI, Sciatica Bothersomeness Index.

After a preliminary eligibility screen on the phone (figure 1), patients will attend a baseline appointment with a clinically trained investigator (eg, physiotherapist) at the local University Department. During the baseline appointment, the diagnosis of sciatica will be confirmed, and the prognostic variables will be assessed through a detailed set of clinical phenotyping as described below. Some patients will also undergo an MRI scan of their lumbar spine. We will then follow-up patients over 1 year with monthly pain diaries (online supplemental appendix 1) and outcome will be measured at 3 (short term) and 12 months (long term). Published sciatica trajectories suggest that most improvement occurs within the first 3–4 months with little change up to 36 months.^20^ Our time points should, therefore, give a comprehensive idea about short and long-term outcome and are similar to other longitudinal sciatica cohorts, thus facilitating cross-comparison.^8^

10.1136/bmjopen-2023-072832.supp1 Supplementary data

### Outcome measures to define pain persistence

The final selection of our outcome measures has been guided by our PAG and feedback from participants in the feasibility study. Pain persistence will be defined with the sciatica bothersomeness index (SBI)^21^ and a Numerical Pain Rating Scale (0 no pain to 10 worst pain imaginable, primary outcomes). The Sciatica Bothersomeness Index (SBI) includes elements of leg pain as well as sensory and motor disturbances, thus providing a comprehensive measure of different sciatica symptoms. This index has shown good discrimination between self-reported successful and non-successful outcome in patients with sciatica^22^ and has been favoured by our PAG. In our feasibility study both outcome measures identified 38% of participants who developed persistent pain, which is in line with previous reports.^9^ In line with recommendations, we will use continuous outcomes for statistical analyses. We may use dichotomisation to help data presentation in figures/tables. In this case, we will use a cut-off of>6.5 on the SBI, which has good validity to identify patients with unsuccessful sciatica outcome.^22^

We may also run analyses using secondary outcomes (eg, disability using Oswestry Disability Index (ODI 2.1a),^23^ self-perceived change using Global Rating of Change Scale.^24^

### Primary mechanism-based prognostic variables

#### Self-reported sensory profiling

See table 1 for questionnaires. The Neuropathic Pain Symptom Inventory and PainDETECT will be used to determine sensory symptom clusters as previously reported.^25^ Patients will be instructed to report the localisation of pain, paraesthesia and hypoesthesia on separate body charts by means of pen-on-paper pain drawings (A4 sheets including ventral and dorsal view of female or male body). All drawings will be digitised and analysed using online software (https://syp.spslab.ch). The derived variables (ie, extent and location) will be used to describe the symptoms associated with sciatica at baseline. These have been shown to provide clues about central sensitisation^26 27^ and may predict clinical outcome in other conditions.^28 29^

**Table 1:** Questionnaires

Questionnaires *primary outcome **secondary outcome		FORECAST patients		Healthy volunteers	Painstorm dataset
		Baseline	Follow-up	Baseline	Extended
FORECAST outcomes	Sciatica Bothersomeness Index (SBI)^21^	X	X*		
	Numerical Pain Rating Scale—previous 2 weeks (worst, least, average for leg /back pain)	X	X*		
	Global Rating of Change Scale		X**		
Neuropathy/neuropathic pain	PainDETECT^79^	X	X		X
	Neuropathic Pain Symptom Inventory (NPSI)^80^	X	X	X	X
	DN4^69^	X	X		X
	Michigan Neuropathy Screening Instrument (MNSI)^70^				X
Pain location, severity	Pain location—list of sites, body chart	X	X	X	X
	Monthly pain diary	X	X		
	Chronic Pain Grade (CPG)^71^				X
	Brief Pain Inventory (BPI)^72^				X
Disability	Oswestry Disability Index (ODI 2.1a)^57^ – Leg Pain	X	X**		
	Oswestry Disability Index (ODI 2.1a)^57^—Low Back Pain	X	X		
	Oswestry Disability Index (ODI 2.1a)^57^—combined leg and back pain			X	
Risk	Keele STarT Back tool^58^	X			
Lifestyle	International Physical Activity Questionnaire (IPAQ, long version)^81^	X	X	X	X
Quality of life	EQ-5D-5L (v1.2)^59^	X	X	X	X
Psychosocial questionnaires	PROMIS SF8a—Ability to participate in social roles and activities^43^ (v1.0)	X	X	X	X
	Pain Catastrophising Scale (PCS)^44^	X	X	X	X
	PROMIS SF8-a—Depression and Anxiety^43^ (v1.0)	X	X	X	X
	Adverse Childhood Events (ACEs) (none, 1, 2,>2)	X		X	X
	Prolonged hospitalisation for life-threatening condition (yes/no)	X		X	X
	PROMIS SF8a—Sleep Disturbance^43^ (v1.0)	X	X	X	X
	PROMIS SF8a—Fatigue^43^ (v1.0)	X	X	X	X
	PROMIS SF4a—instrumental support^43^ (v1.0)	X	X	X	X
	PROMIS SF4a—Emotional Support^43^ (v1.0)	X	X	X	X
	Ten Item Personality Index (TIPI)^45^	X		X	X
	State Optimism Measure (SOM-7)	X	X	X	X
	Illness Perception Questionnaire (IPQ-R)^82^	X			
	Sciatica Perception Questionnaire (SPQ)	X	X		
	Stigma Scale for Chronic Illnesses (SSCI)—modified^48^	X	X		
	‘in your own words’ (impact on social and financial situation)				X

FORECAST: Factors predicting the transition from acute to persistent pain in people with ‘sciatica’

#### Somatosensory profiling

There is preliminary evidence that some quantitative sensory testing (QST) parameters may be prognostic in patients with a range of pain conditions including neuropathic pain.^15 16^ The standardised and validated QST battery developed by the German Network for Neuropathic Pain (Deutscher Forschungsverbund Neuropathischer Schmerz, DFNS) will be used to reliably determine sensory function in different nerve fibres. Cold and warm detection thresholds (CDT, WDT; average of three repetitions) as well as cold and heat pain thresholds (CPT, HPT, average of three repetitions) and thermal sensory limen including paradoxical heat sensations during three series of alternating cold and warm stimuli will be examined with a Thermotester (Somedic, Sweden, 25×50 mm thermode). Mechanical detection thresholds will be measured with von Frey hairs and mechanical pain thresholds (MPT) with weighted pin-prick stimulators (geometric mean of five series of ascending and descending stimuli). Mechanical pain sensitivity will be examined with a Numerical Pain Rating Scale (0–100) during a shortened protocol of two sets of seven pseudo-random pin-prick stimulations.^30^ To determine the presence of allodynia, two sets of three light touch stimulations with a cotton wisp, a cotton wool tip and a standardised brush (Sense-lab) will be intermingled with these pin-prick stimulations. Pressure pain thresholds (PPT) will be evaluated with a manual algometer (Wagner Instruments, USA) and vibration detection threshold with a Rydel Seiffer tuning fork (average of three repetitions). The wind-up ratio will be determined as the mean numerical pain rating of three trains of 10 pin-prick stimuli divided by the mean rating of three single stimuli.

A shortened QST battery will first be conducted on the hand ipsilateral to the (most) symptomatic leg (CPT, HPT and MPT on dorsum of hand; PPT over thenar eminence) to determine the presence of widespread hyperalgesia. The full QST protocol will then be performed in the area of maximal pain in the affected leg where pervious work has shown QST changes in patients with ‘sciatica’.^31^

We will use healthy control data to calculate Z-scores, where each individual parameter is related to its region-specific, age-specific and gender-specific reference range. We will collect our own normative data, assisted by the provision of an existing QST dataset.^32^ Using a previously published algorithm,^13^ patients will also be assigned one of the following somatosensory profiles (1) sensory loss, (2) thermal hyperalgesia, (3) mechanical hyperalgesia.

Furthermore, we will include a conditioned pain modulation (CPM) paradigm to examine the efficacy of the descending pain modulatory system. Such dynamic QST protocols have shown most promising prognostic ability in other pain conditions.^15 16^ Based on current recommendations,^33^ we will evaluate a sequential CPM paradigm using PPT over the thenar eminence of the dominant hand (test stimulus, average of three repetitions) and cold-water immersion of the non-dominant hand to the level of the wrist (conditioning stimulus). This combination has provided the most reliable and large magnitude CPM effects.^34^ The water bath will be standardised to 4°C±2°C by adding ice. Patients are asked to report the intensity of pain experienced by cold water immersion from 0 (no pain) to 100 (worst pain imaginable). Once the pain reaches the cut-off of >40/100, or after a maximum of 2 min if this cut-off is not reached,^33 35^ the participants will be asked to remove the hand from the water bath. The test stimulus will be repeated immediately thereafter. Cold water immersion is the most used CPM conditioning stimulus and is easy to implement and seems to be the most effective CPM paradigm.^36 37^ PPT measurements are convenient, quickly measured and frequently used as a test stimulus.^38^ A good to excellent intrasession reliability for CPM assessment with PPTs has been reported.^37 39^

#### Psychosocial profiles

There is a large body of evidence supporting the role of psychosocial factors in the persistence of pain and disability.^40 41^ Therefore, we will assess psychosocial factors to examine their prognostic value in sciatica. The selection of specific measures of psychosocial factors drew on existing evidence for their predictive utility in the context of other pain conditions, their theoretical relevance and their psychometric properties including content validity.^42^ We will have a two-level approach to assessment that includes general or ‘transdiagnostic’ psychosocial factors and condition/sciatica-specific factors (table 1). The transdiagnostic factors include symptoms of depression and general anxiety, sleep disturbance and fatigue (all measured with their respective PROMIS SF8a tools,^43^ trauma history, pain-related worry (‘Pain Catastrophizing Scale’)^44^ and personality (Ten Item Personality Inventory).^45^ In addition to transdiagnostic psychosocial risk factors, we have included several measures of potential protective factors (ie, optimism, State Optimism Measure^46^; social support, PROMIS SF4a instrumental and emotional Support and social role participation, PROMIS SF8a) to provide a more holistic assessment. To assess cognitions specific to the context of sciatica, we developed a novel item set that was primarily adapted from the revised Illness Perception Questionnaire (online supplemental appendix 2).^47^ Patient partners provided extensive feedback to develop and refine the sciatica-specific adaptation of these items. We have also included a measure of stigma^48^ in relation to sciatica.

#### Blood inflammatory markers

We will sample blood by cubital venepuncture into BD Vacutainer SST and serum clot activator tubes (gold and red cap, BD, Wokingham United Kingdom). The time of last meal will be recorded. Thirty minutes after venepuncture, the blood will be centrifuged at 1.3 g for 10 min at 4°C (gold cap for protein analysis) and at room temperature (red cap tubes for metabolomics). The serum fraction will be immediately aliquoted and stored at −80°C for batch processing.

We will use complimentary protein/metabolomics analysis to evaluate serum inflammatory markers related to inflammation and neuropathic pain. Protein analysis will usecustom-made electrochemiluminescent multiplex biomarkers assays. These plates contain 17 cytokines/chemokines including candidates of interest derived in our previous work (eg, IL-4, IL-9, IL-6).^49^ Patient samples will be run in duplicate and normalised to standard curves.

Metabolomic analyses will be carried out using a state-of-the-art, high-field 700 MHz NMR spectrometer equipped with TCI cryoprobe (Department of Chemistry, University of Oxford), as previously described.^50^ Quality control samples will be randomly spread throughout the run for standardisation and internal reference standards will allow absolute concentrations of inflammatory markers (N-acetylated glycoprotein species, serum lipoproteins) along with energy and tricarboxylic acid cycle

### Additional phenotypic data

#### Demographi and medical information

We will also collect basic demographic data (eg, age, gender, ethnicity, profession, working status, perception of household income, years of school attendance) and medical information (eg, most affected side, previous history of back pain or sciatica, number of previous episodes, duration of current episode, family history of pain, current and past medical history including current and previous medications and their effectiveness, trialled treatments, results of previous imaging, smoking and alcohol intake, online supplemental appendix 3).

#### Clinical examination

We will also perform a clinical examination (online supplemental appendix 4). We will document height, weight and hip/waist circumference. We will record findings from a bedside neurological screening examination of the lower limbs. This includes myotomal testing from lumbar levels L2-S1, patellar and achilles tendon reflexes as well as mapping of sensory loss to light touch and pin prick on body charts. We will check for upper motor neuron signs (exclusion criteria) using Hoffmann’s test, Babinski, inverted supinator sign and observation of tandem walk.^51^ Patients will go through a warning sign checklist for suspected cauda equina syndrome (exclusion criteria).^52^

We will perform the straight leg raise and slump test as well as femoral slump if indicated (eg, presentation suggesting upper lumbar involvement).^53^ These tests for nerve mechanosensitivity will be deemed positive if they (1) reproduce at least partially the patients’ symptoms and (2) if structural differentiation through either foot dorsiflexion or cervical flexion changes the symptoms.^54^ We will further record the presence of lumbar shifts, active range of motion restrictions in lower back and hip including whether these movements provoke back or leg symptoms. Pain provocation on posterior anterior intervertebral movement palpation of the lumbar segments L1-L5 will be recorded (Grade IV unless pain provocation occurs earlier).

At the end of the baseline appointment, the assessor will rate the certainty of neuropathic leg pain as unlikely, possible, probable or definite according to the updated neuropathic pain grading system.^55^ They will also assign patients to one or several of the following subgroups described elsewhere^56^: radiculopathy (true neurological deficit), radicular pain, neural mechanosensitivity or somatic referred pain.

#### Self-reported questionnaires

We will also collect the following additional questionnaires to describe our patient population: ODI^57^ (separate questionnaires for back and leg), Keele Start Back tool,^58^ EQ-5D^59^ and a monthly pain diary (online supplemental appendix 3).

#### Magnetic resonance neurography

We will perform magnetic resonance neurography (MRN) in a subset of n=100 patients with sciatica and n=44 healthy matched controls to identify moderate effects^23^ (d=0.52, alpha=0.05, 80% power). Eligible patients (eg, MRI safety) will be consecutively recruited for scanning until numbers are reached.

We will perform advanced MRN optimised to visualise lumbar nerve root macrostructure and microstructure at 3 Tesla using a dedicated 18-channel phased array spine coil (Siemens, UK). The protocol includes multishell (b=700 and 1500 s/mm^2^) diffusion tensor imaging (DTI) scans, high-resolution anatomic scans with optimised T1 and T2-weighted contrasts, and a T2 mapping scan (DOI: 10.5281/zenodo.7760905). The data analysis will be performed using functional magnetic resonance imaging of the brain software library (FSL) tools including TOPUP^60 61^ and EDDY^62–64^ for the correction of images’ distortions and subject movements, DTIFIT^65^ for the fitting of diffusion tensor model and FLIRT^66 67^ for the registration of diffusion metrics and anatomic images. Measures including fractional anisotropy, mean/axial/radial diffusivity and T2 maps will be obtained within regions of interest in lumbar nerve roots (affected and unaffected sides) and averaged over multiple slices as we have optimised before.^68^

### Cohort harmonisation

The FORECAST cohort is harmonised with the Advanced Pain Discovery Platform funded PAINSTORM consortium, and, therefore, includes additional measures that will allow data integration (eg, blood collection for genetic analyses, skin biopsies in the maximal pain area, DN4,^69^ Michigan Neuropathy Screening Instrument,^70^ Chronic pain grade,^71^ Brief pain inventory^72^ (pain intensity items), a section where patients can tell us more about their pain and circumstances in their own words including how they would describe their pain to their friends/family or work colleagues as well as their feelings about their financial situation and its impact on their situation. This harmonisation may also enable external validation of the FORECAST findings in other neuropathies.

### Data analysis plan

Statistical methods will follow STROBE guidelines^17^ and the TRIPOD statement for ransparent reporting of a multivariable prediction model for individual prognosis or diagnosis.^18^

Participants’ baseline characteristics (eg, demographics, pain severity, disability, medical comorbidities) and their clinical course (primary and secondary outcomes, ODI) will be described for short (3 months) and long-term time points (12 months).

To identify and characterise mechanism-based subgroups in patients with acute/subacute sciatica and use distance-based clustering algorithms efficiently we first need to address the high dimensionality—modest sample size of the data set. Thus, we will first carry out a principal component analysis to summarise and reduce the dimensionality of the dataset while preserving as much variability as possible. Then we will use algorithmic centroid (k-means) and hierarchical clustering based on the Euclidean distance between principal dimensions to identify subgroups of patients sharing high phenotypic similarities. The optimal number of clusters will be determined using the gap statistic and the elbow of the within/between clusters variance plot. Consequently, we will perform hypothesis testing to assess group differences on the original variables between participants assigned to different clusters. All omnibus tests will be followed up by the appropriate post hoc test.

To investigate factors that predict pain persistence in people with sciatica, we will use variable selection techniques followed by predictive modelling. First, we will perform filtering of the original variables by calculating the univariate associations (coefficients, 95% CI, p values) between variables and the outcome and between each other. We will select a subset of uncorrelated variables that are associated with the outcome and use them as input features in machine learning algorithms for high-dimensional, small data sets that will allow us to identify the most powerful predictors and assess model selection/predictive accuracy. During preprocessing, missing data will be examined, the mechanism of missingness will be inferred using hypothesis testing and visually assessed using a matrix of boxplots for all pairs of variables and the outcome, and, if appropriate, multiple imputation by chained equations will be used. Drawing from machine learning techniques for high-dimensional small data sets, we will use resampling and validation in the form of repeated cross-validation to perform a complete variable profiling to identify the most powerful predictors. Multivariate adaptive regression splines (MARS) with built-in feature selection and Decision Tree models known to work well on low sample sizes will be trained to predict the 3-month and 1-year outcome. Model performance will be estimated using five times repeated 10-fold cross-validation and compared with models trained on surrogate data.^73^ The latter benchmarking technique is appropriate for small data sets, where holding out a subset of data before the analysis to be used as a pseudo-independent test set is impossible. Instead, an artificial—surrogate data set, preserving the descriptive statistics but not any of the potentially real associations between the variables and the outcome of the original dataset, will be created and the performance of models trained on the actual and surrogate dataset will be compared. Models’ predictive performance will be reported alongside variable importance rankings. Model selection will be done to maximise the Mathews correlation coefficient for dichotomised outcomes and to minimise the root mean square error for continuous outcomes during cross-validation. Scalar metric estimations of predictive performance including accuracy (binomial test p value against the majority class prevalence), balanced accuracy and the area under the precision/recall curve will be reported alongside their 95% CI. Predictor importance will be assessed using model specific techniques, that is, the reduction in performance estimated by cross-validation when each predictor is removed for MARS and node impurity for tree-based methods. Variables’ influence on the predicted outcome both at the global and individual level will be quantified by the partial dependence plots and individual conditional expectation,^74^ respectively. These will show the average marginal effect on the prediction given a certain value of a predictor variable and provide model interpretability.

### Sample size estimation

#### QST sensory profiles

Published sample size guidelines for QST clustering in peripheral nerve injury^75^ suggest that for strong effects (effect size=0.7) a sample size of <180 patients will produce a subpopulation with thermal and mechanical hyperalgesia large enough to conduct a study with 80% power, at an alpha 0.05. To calculate QST z-scores, at least eight controls are required for each area and age decade.^76^ Our feasibility study included patients of 7 age decades with three main pain areas. We will, therefore, need n=168 controls.

#### K-means and hierarchical clustering after PCA

Using the two first principal dimensions for three variable domains (self-reported profiling, QST, inflammatory markers), we will need 2^6^=64 patients to perform k-means clustering with adequate power.^77^

#### Algorithmic cluster analysis

Assuming k=4 clusters, we will be able to identify moderate effects (effect size=0.25) with an one-way analysis of variance between four groups at an alpha level of 0.05, 80% power.

#### Predictor profiling

FORECAST aims to identify prognostic factors (the first step in the PROGRESS framework)^78^ rather than developing a clinical prognostic tool or individual risk model which requires much larger sample sizes. We will use robust algorithms that include feature selection, and we will assess model performance using methods developed for small data sets and robust metrics. As this part is an exploratory analysis that could shape future hypotheses and validation studies, our sample size is adequate. Given the anticipated sample size ratios with chronic (180×30%=54) and resolved sciatica (180×70%=126) and accounting for 15% attrition (see feasibility study), we will be able to identify moderate effects (effect size=0.5) using a two-tailed Wilcoxon-Mann-Whitney test (power 81%, alpha 0.05).

## Ethics and dissemination

The FORECAST study has received ethical approval (South Central Oxford C, 18/SC/0263). All participants will provide informed written or electronic consent before participating in the study.

The dissemination strategy will be strongly guided by our PPIE activities (see below). This will be based on coproductions between patient partners and academics and will involve publication of findings in scientific journals, presentations at conferences, media pieces (mainstream and social media) as well as communication through charity partners.

Data will be made publicly available on the ALLEVIATE data hub (https://alleviate.ac.uk) and remaining bio-samples will be on-boarded to the Imperial Biobank. The data and samples will continue to be linked and will be available for future studies.

## PPIE and dissemination of findings

The FORECAST team consists of equal partners including patient partners, clinicians and researchers. Our aims have been shaped by the needs of people living with sciatica to ensure we address unmet needs. The PPIE plans will be shaped by the following members of FORECAST: (1) inclusion of two patient partners as coinvestigators (CR and CP). They will contribute as equal partners on the investigator team; (2) PPIE lead with extensive experience in involving patients’ voices in research (KRM); (3) diverse PAG consisting of six individuals with a lived experience of sciatica. Our patient partners and PAG provided early input to the original grant application and identification of key research activities within the project, particularly around including the feasibility work (eg, acceptability of testing and study procedures), study design (eg, selection of primary outcome measure) and strongly informed the writing of our funding application (eg, lay summary).

We will continue to work closely with people with lived experience of sciatica as we undertake this study, and our PPIE strategy will continue to be implemented throughout the lifetime of FORECAST. We will seek the perspective and guidance of our patient partners and PAG members on matters including, but not limited to participant recruitment and retention; barriers/facilitators of participation among seldom heard populations; data analysis and sensemaking of findings, organisation and coproduction of workshops, dissemination materials and public engagement activities. This will ensure that the patient perspective has been considered at all stages throughout the project.

We will also work closely with our patient partners and advisors on engagement and dissemination activities. This may include, but is not limited to, coproducing newsletters, lay summaries, website content, infographics, animated videos, and podcasts, as well as engagement activities to bring the project into a public sphere. We plan to work closely with the PAINSTORM research team and patient partners as well as other national and international pain and sciatica groups to promote the study and its subsequent findings. This would allow us to reflect on the way the conclusions are presented and identify any gaps which might lead to further research in the topic area. We also plan to hold conversations with our patient partners and PAG regarding planning and undertaking academic dissemination activities (eg, engagement with policy stakeholders, conference abstracts/presentations, manuscript preparation/publication). All individuals who contribute to this PPI advisory group will receive payment in accordance with current INVOLVE guidelines.

## Supplementary Material

Reviewer comments

Author's manuscript
