# Supplementary material for: Factors predicting the transition from acute to persistent pain in people with ‘sciatica’: the FORECAST longitudinal prognostic factor cohort study protocol
Source: BMJ Open. 2023 Apr 5;13(4):e072832. doi: 10.1136/bmjopen-2023-072832 (PMC10111910; doi:10.1136/bmjopen-2023-072832)
Supplement: Supplementary data [file bmjopen-2023-072832supp001.pdf]

Appendix 1: Pain trajectory Diary

ID______________
Date______________

Pain Trajectory Diary - FORECAST

Month:

Thank you for your continuing support of the FORECAST study. Please let us know below about your sciatica pain in the past 2 weeks.

	No pain 0	1	2	3	4	5	6	7	8	9	Worst pain imaginable 10
Sciatica leg pain											
**In the last two weeks, at its worst, how intense was your sciatica leg pain?**											
**In the last two weeks, at its least, how intense was your sciatica leg pain?**											
**In the last two weeks, on average, how intense was your sciatica leg pain?**											
Low back pain											
**In the last two weeks, on average, how intense was your back pain?**											

Appendix 2: Sciatica Perception Questionnaire

Your views about your sciatica (SPQ)

We are interested in your own personal views of how you currently see your sciatica.

Please indicate how much you agree or disagree with the following statements about your sciatica by ticking the appropriate box.

	Strongly disagree	Disagree	Neither agree nor disagree	Agree	Strongly agree
I expect that I am going into old age with my sciatica					
I feel that my sciatica will last for a long time					
My sciatica is likely to be permanent rather than temporary					
I expect that the effect of my sciatica on day-to-day life will worsen over time					
My sciatica comes and goes					
I do not know how my sciatica will change in the future					
My sciatica is a burden to others					
My sciatica can put me in awkward and embarrassing situations					
I have the personal strength to manage my sciatica					
I avoid specific positions and/or movements due to fear of causing pain					
I avoid specific positions and/or movements due to fear of causing damage					
There is little that I can do to improve my sciatica myself					
There is something seriously wrong with my back/leg					
The cause of my sciatica has not been investigated properly					
I am concerned about possible adverse long term consequences of the treatment for my sciatica					
There is nothing that can help my sciatica					
I do not understand what is wrong with my back/leg					
My current treatment does not make sense to me					
I worry that I am not getting the right treatment for my sciatica					
I don’t know what activities I can safely do with my sciatica					
It is so unfair that I have sciatica					

Appendix 3: Demographic and Medical History Data

Demographics	FORECAST substudy		Healthy Volunteers	PAINSTORM DATASET
	BASELINE	FOLLOW UP	BASELINE	EXTENDED
Age (yrs)	X		X	X
Sex	X		X	X
Years in education	X		X	X
Working status*	X		X	X
Household income**	X		X	X
Ethnicity	X		X	X
**Medical history**				
History of sciatica (date of first episode, number of previous episodes)	X			
Duration of current sciatica episode (days)	X			
Is the leg pain worse than the back pain?	X			
Affected leg (left/right/both)	X			
Family history of chronic pain	X		X	
Details of other medical diagnoses	X		X	
Cauda equina screening questions	X			
Types of treatments received for sciatica to date	X	X		
Types of tests/ investigations undertaken for sciatica to date	X	X		
Relevant previous and current medication, including whether or not they are taken for sciatica	X	X	X	
Medications: efficacy, adherence	X	X	X	X
Tobacco and Alcohol intake	X		X	X

* Working status:

- ☐ In paid employment or self-employed
- ☐ Retired
- ☐ Looking after home and/or family
- ☐ Unable to work because of sickness or disability
- ☐ Unemployed
- ☐ Doing unpaid or voluntary work
- ☐ Full or part-time student
- ☐ None of the above
- ☐ Prefer not to answer

** Which of the descriptions below comes closest to how you feel about your household's income nowadays?

- ☐ Living comfortably on present income
- ☐ Coping on present income
- ☐ Finding it difficult on present income
- ☐ Finding it very difficult on present income
- ☐ Do not wish to answer
- ☐ Don't know

Appendix 4: Clinical Examination

**Clinical Examination**<br>**(Identical for people with sciatica and healthy volunteers apart from assessments indicated with *which are performed only on people with sciatica)**	
Height (cm)	
Weight (kg)	
Waist circumference (cm)	
Hip circumference (cm)	
Myelopathy screening cluster	Tandem gait, inverted supinator sign, Hoffman's test, Babinski reflex
Neural mechanosensitivity*	straight leg raise, slump, femoral slump (where clinical picture indicates). Rated as negative or positive (at least partial symptom reproduction plus structural differentiation changes symptoms).
Lumbar spine active range of motion	flexion, extension, bilateral side flexion.<br>Range recorded as full or restricted.<br>Symptom provocation recorded as: none, leg, back, leg + back.
Palpation of lumbar spine*	Passive accessory intervertebral mobilisations (PAIVMS) over spinous processes L1-L5 centrally (to end of resistance if required).<br>Symptom provocation recorded as none, leg, back, leg + back
