# Supplementary material for: Factors predicting the transition from acute to persistent pain in people with ‘sciatica’: the FORECAST longitudinal prognostic factor cohort study protocol
Source: BMJ Open. 2023 Apr 5;13(4):e072832. doi: 10.1136/bmjopen-2023-072832 (PMC10111910; doi:10.1136/bmjopen-2023-072832)
Supplement: Author's manuscript [file bmjopen-2023-072832.draft_revisions.pdf]

BMJ Open is committed to open peer review. As part of this commitment we make the peer review history of every article we publish publicly available.

When an article is published we post the peer reviewers' comments and the authors' responses online. We also post the versions of the paper that were used during peer review. These are the versions that the peer review comments apply to.

The versions of the paper that follow are the versions that were submitted during the peer review process. They are not the versions of record or the final published versions. They should not be cited or distributed as the published version of this manuscript.

BMJ Open is an open access journal and the full, final, typeset and author-corrected version of record of the manuscript is available on our site with no access controls, subscription charges or pay-per-view fees (http://bmjopen.bmj.com).

If you have any questions on BMJ Open's open peer review process please email info.bmjopen@bmj.com

BMJ Open

Factors predicting the transition from acute to persistent pain in people with 'sciatica'-the FORECAST longitudinal prognostic factor cohort

Journal:	*BMJ Open*
Manuscript ID	bmjopen-2023-072832
Article Type:	Protocol
Date Submitted by the Author:	15-Feb-2023
Complete List of Authors:	Schmid, Annina B.; University of Oxford Nuffield Department of Clinical Neurosciences<br>Ridgway, Lucy; University of Oxford Nuffield Department of Clinical Neurosciences<br>Hailey, Louise; University of Oxford Nuffield Department of Clinical Neurosciences; University of Oxford Nuffield Department of Orthopaedics Rheumatology and Musculoskeletal Sciences<br>Tachrount, Mohamed; University of Oxford Nuffield Department of Clinical Neurosciences<br>Probert, Fay; University of Oxford Department of Chemistry<br>Martin, Kathryn; University of Aberdeen, Epidemiology Group, Institute of Applied Health Sciences<br>Scott, Whitney; King's College London,<br>Crombez, Geert; University of Ghent,<br>Price, Christine; University of Oxford Nuffield Department of Clinical Neurosciences<br>Robertson, Claire; University of Oxford Nuffield Department of Clinical Neurosciences<br>Koushesh, Soraya; University of Oxford Nuffield Department of Clinical Neurosciences<br>Ather, Sarim; Oxford University Hospitals NHS Foundation Trust<br>Tampin, Brigitte; Sir Charles Gairdner Hospital, Physiotherapy; Curtin School of Allied Health<br>Barbero, Marco; University of Applied Sciences and Arts of Southern Switzerland<br>Nanz, Daniel; Balgrist University Hospital, Swiss Centre for Musculoskeletal Imaging, Balgrist Campus<br>Clare, Stuart; University of Oxford Nuffield Department of Clinical Neurosciences<br>fairbank, jeremy; Nuffield Orthopaedic Centre, orthopaedic surgery<br>Baskozos, Georgios; University of Oxford, Neural Injury Group, Nuffield Department of Clinical Neuroscience, John Radcliffe Hospital
Keywords:	Chronic Pain, Neurology < INTERNAL MEDICINE, Rehabilitation medicine < INTERNAL MEDICINE, Rheumatology < INTERNAL MEDICINE, Neurological injury < NEUROLOGY, Neurological pain < NEUROLOGY

SCHOLARONE™
Manuscripts

Factors predicting the transition from acute to persistent pain in people with 'sciatica'-the FORECAST longitudinal prognostic factor cohort

Annina B Schmid[1,2], Lucy Ridgway[1], Louise Hailey[1,15], Mohamed Tachrount[1,2], Fay Probert[3], Kathryn R Martin[4,5], Whitney Scott[6,7], Geert Crombez[8], Christine Price[9], Claire Robertson[9], Soraya Koushesh[1], Sarim Ather[10], Brigitte Tampin[11,12,13], Marco Barbero[14], Daniel Nanz[15], Stuart Clare[1,2], Jeremy Fairbank[16], Georgios Baskozos[1]

[1] Nuffield Department of Clinical Neurosciences, University of Oxford, Oxford, UK

[2] Wellcome Centre for Integrative Neuroimaging, University of Oxford, Oxford, UK

[3] Department of Chemistry, University of Oxford, UK

[4] Academic Primary Care, Institute of Applied Health Sciences, School of Medicine, Medical Sciences and Nutrition, University of Aberdeen, UK

[5] Aberdeen Centre for Arthritis and Musculoskeletal Health, School of Medicine, Medical Sciences and Nutrition, University of Aberdeen, UK

[6] King's College London, Health Psychology Section, Institute of Psychiatry, Psychology, and Neuroscience

[7] INPUT Pain Management Unit, Guy's and St Thomas' NHS Foundation Trust, London, UK

[8] Department of Experimental-clinical and Health Psychology, Ghent University, Ghent, Belgium

[9] FORECAST Patient Partners

[10] Oxford University Hospital NHS Foundation Trust, Oxford, UK

[11] Department of Physiotherapy, Sir Charles Gairdner Hospital, Perth, Australia

[12] Curtin School of Allied Health, Faculty of Health Sciences, Curtin University, Perth, Australia

[13] Faculty of Business and Social Sciences, Hochschule Osnabrueck, University of Applied Sciences, Osnabrueck, Germany

[14] Rehabilitation Research Laboratory 2rLab, Department of Business Economics, Health and Social Care, University of Applied Sciences and Arts of Southern Switzerland, Manno, Switzerland

[15] Swiss Center for Musculoskeletal Imaging, Balgrist Campus AG and Medical Faculty, University of Zurich, Switzerland

[16] Nuffield Department of Orthopaedics, Rheumatology and Musculoskeletal Sciences, University of Oxford

Corresponding author:

Annina B Schmid

Nuffield Department of Clinical Neurosciences

John Radcliffe Hospital

West Wing Level 6

For peer review only

Headley Way
OX39DU, Oxford, UK
Phone: +44 (0)1865 223254
Email: annina.schmid@neuro-research.ch
https://www.ndcn.ox.ac.uk/team/annina-schmid

word count: 4258

For peer review only

Abstract

Introduction: Sciatica is a common condition and is associated with higher levels of pain, disability, poorer quality of life, and increased use of health resources compared to low back pain alone. Although many patients recover, a third develop persistent sciatica symptoms. It remains unclear, why some patients develop persistent sciatica as none of the traditionally considered clinical parameters (e.g., symptom severity, routine magnetic resonance imaging) are consistent prognostic factors. The FORECAST study will take a different approach by exploring mechanism-based subgroups in patients with sciatica and investigate whether a mechanism-based approach can identify factors that predict pain persistence in patients with sciatica.

Methods and analysis. We will perform a prospective longitudinal cohort study including 180 people with acute/subacute sciatica. N=168 healthy participants will provide normative data. A detailed set of variables will be assessed within 3 months after sciatica onset. This will include self-reported sensory and psychosocial profiles, quantitative sensory testing, blood inflammatory markers and advanced neuroimaging. We will determine outcome with the sciatica bothersomeness index and a numerical pain rating scale for leg pain severity at 3 and 12 months.
We will use principal component analysis followed by clustering methods to identify subgroups. Univariate associations and machine learning methods optimised for high dimensional small datasets will be used to identify the most powerful predictors and model selection/accuracy.
The results will provide crucial information about the pathophysiological drivers of sciatica symptoms and may identify prognostic factors of pain persistence.

Ethics and dissemination: The FORECAST study has received ethical approval (South Central Oxford C, 18/SC/0263). The dissemination strategy will be guided by our patient and public engagement activities and will include peer-reviewed publications, conference presentations, social media and podcasts.

Registration: ISRCTN18170726

Keywords: sciatica, radiculopathy, radicular pain, prognosis, neuropathic pain

For peer review only

Article summary

Strength and limitations

- This study has the potential to advance our understanding of the heterogeneity of pathomechanisms in people with sciatica and to identify factors that predict pain persistence.
- This dataset will include the largest deeply phenotyped 'sciatica' cohort to date.
- Harmonisation with the PAINSTORM consortium will afford integration of the FORECAST cohort into a much larger dataset of neuropathic pain.
- The large amount of data points collected for a modest cohort size will pose challenges for analyses and will require dimensionality reduction techniques
- Patient recruitment will be challenging given the time intensive phenotyping protocol. This may lead to recruitment bias.

For peer review only

Introduction

Low back pain (LBP) is associated with more disability than any other condition.[1] Up to 60% of patients with LBP also experience leg pain, which is associated with worse health outcomes. In some cases, the leg pain is caused by nerve root involvement, commonly referred to as 'sciatica'. Whereas some patients with 'sciatica' have pain of predominantly nociceptive character, others develop neuropathic (nerve related) pain, which is characterised by burning pain, electric shocks or tingling. The presence of neuropathic pain in sciatica further increases suffering and disability.[2] The management of sciatica is therefore a priority. The NICE guidelines recommend a period of non-invasive treatment (e.g., medication, physiotherapy) before invasive treatment (e.g., surgery) is considered.[3] Sadly, first line management for patients with sciatica remains largely ineffective[4 5] and at least one third develops persistent pain and disability lasting a year or longer.[6-10]

It remains unclear why some patients develop persistent sciatica. Two recent systematic reviews have established that none of the traditionally considered clinical parameters (e.g. pain intensity, routine magnetic resonance imaging [MRI], mental wellbeing) are consistent prognostic factors.[11 12] Since those publications, the largest prognostic study in patients with sciatica in primary care[8] identified several factors that are weakly associated with improvement, these included shorter pain duration, belief that symptoms will not last long, myotomal weakness, overall impact of sciatica. However, at 12 months, only two factors were independently associated with outcome in the multivariable model analysis. This restricts the usefulness of predictive modelling for risk estimation of outcome for individual patients. The absence of prognostic factors hinders the early identification of patients at risk of developing persistent pain and prevents personalised treatments.

These challenges in management and risk prediction are partly attributed to a lack of understanding of the pathomechanisms at play in sciatica. Sciatica is a heterogeneous condition likely caused by differing mechanisms in individual patients,[13] which are potentially amenable to targeted treatment. In the field of neuropathic pain, mechanism-based stratification using deep phenotyping has been advocated to facilitate personalised pain management.[14] In contrast to traditionally used methods that quantify the severity of the disease with a limited battery of basic clinical measures (e.g., routine MRI scans, symptom severity basic questionnaires), a mechanism-based approach aims to stratify patients by the distinct underlying mechanisms. It has been suggested that the nature of the pathomechanisms at play in patients with pain may influence treatment outcome and prognosis.[14-16] The utility of such a mechanism-based approach in predicting pain persistence in people with sciatica remains unknown.

For peer review only

The FORECAST study will examine the value of a mechanism-based deep phenotyping approach including main domains assessing nerve function, nerve structure, inflammation and psychosocial factors.

The aims of the FORECAST study are:

1. To explore mechanism-based subgroups in patients with acute/subacute sciatica.
2. To investigate whether a mechanism-based approach can identify factors that predict pain persistence in people with sciatica.

Methods

The FORECAST study is a prospective longitudinal prognostic factor cohort study that is based on feasibility data and closely informed by patient and public involvement and engagement (PPIE) activities including feedback from our named patient partners, six-member patient advisory group, and survey results from participants of the feasibility study. The study will be performed and reported according to the guidance for observational studies (STROBE)[17] and the statement for transparent reporting of a multivariable prediction model for individual prognosis or diagnosis (TRIPOD).[18]

Participants

We will include n=180 patients with acute/subacute 'sciatica' and n=168 healthy age and gender matched participants without symptoms of sciatica/low back pain. Healthy participants are important to establish normative values for blood markers, somatosensory profiling and neuroimaging.

People aged >18 years with a clinical diagnosis of 'sciatica' will be recruited from primary care in Oxfordshire (e.g., primary care NHS providers as well as GP, Physiotherapy, Osteopathy and Chiropractor clinics) and through leaflets on public noticeboards. Sciatica symptom onset of the current episode needs to be within the past three months with a symptom free period of at least 3 months preceding the current sciatica symptoms. The inclusion criteria for patients with 'sciatica' are based on a published diagnostic model[19] which includes 5 weighted parameters (self-reported sensory changes, below knee pain, leg pain worse than back pain, neurodynamic tests, neurological deficit). A sum score >4 will be defined as sciatica, with a mean predicted probability of 83%. In addition, patients with suspected sciatica will undergo a clinical examination by a physiotherapist to further confirm the diagnosis of sciatica and rule out other diagnoses (see additional phenotypic data below).

The following exclusion criteria will apply; presence of other nerve-related disorders (e.g. diabetic neuropathy, stroke), previous lumbar spine surgery, serious spinal diseases (e.g. infection, cauda equina syndrome, metastatic lesions), chronic inflammatory disorders, other pain conditions that may confound

For peer review only

assessment (e.g., fibromyalgia), pregnancy, insufficient command of the English language to obtain consent/complete questionnaires, and contraindications to MRI for those selected for scanning.

Study procedure

After a preliminary eligibility screen on the phone (Figure 1), patients will attend a baseline appointment with a clinically trained investigator (e.g, physiotherapist) at the local University Department. During the baseline appointment, the diagnosis of sciatica will be confirmed, and the prognostic variables will be assessed through a detailed set of clinical phenotyping as described below. Some patients will also undergo an MRI scan of their lumbar spine. We will then follow up patients over 1 year with monthly pain diaries (Appendix 1) and outcome will be measured at 3 (short-term) and 12 months (long-term).

Outcome measures to define pain persistence

The final selection of our outcome measures has been guided by our patient advisory group and feedback from participants in the feasibility study. Pain persistence will be defined with the Sciatica Bothersomeness Index[20] and a numerical pain rating scale (0 no pain to 10 worst pain imaginable, primary outcomes). The Sciatica Bothersomeness Index includes elements of leg pain as well as sensory and motor disturbances, thus providing a comprehensive measure of different sciatica symptoms. This index has shown good discrimination between self-reported successful and non-successful outcome in patients with sciatica[21] and has been favoured by our patient advisory group. In our feasibility study both outcome measures identified 38% of participants who developed persistent pain, which is in line with previous reports.[9]

We may also run analyses using secondary outcomes (e.g., disability using Oswestry Disability Index (ODI 2.1a)[22], self-perceived change using global rating of change scale (GROC)[23]).

Primary mechanism-based prognostic variables

1) Self-reported sensory profiling

See Table 1 for questionnaires. The Neuropathic Pain Symptom Inventory (NPSI) and PainDETECT will be used to determine sensory symptom clusters as previously reported.[24] Patients will be instructed to report the localisation of pain, paraesthesia and hypoesthesia on separate body charts by means of pen-on-paper pain drawings (A4 sheets including ventral and dorsal view of female or male body). All drawings will be digitised and analysed using online software (https://syp.spslab.ch). The derived variables (i.e. extent and location) will be used to describe the symptoms associated with sciatica at the baseline. These have been shown to provide clues about central sensitisation[25 26]and may predict clinical outcome in other conditions.[27 28]

2) Somatosensory profiling

For peer review only

There is preliminary evidence that some quantitative sensory testing (QST) parameters may be prognostic in patients with a range of pain conditions including neuropathic pain.[15 16] The standardised and validated QST battery developed by the German Network for Neuropathic Pain (DFNS) will be used to reliably determine sensory function in different nerve fibres. Cold and warm detection thresholds (CDT, WDT; average of three repetitions) as well as cold and heat pain thresholds (CPT, HPT, average of three repetitions) and thermal sensory limen (TSL) including paradoxical heat sensations during three series of alternating cold and warm stimuli will be examined with a Thermotester (Somedic, Sweden, 25x50mm thermode). Mechanical detection thresholds (MDT) will be measured with von Frey hairs and mechanical pain thresholds (MPT) with weighted pin-prick stimulators (geometric mean of five series of ascending and descending stimuli). Mechanical pain sensitivity (MPS) will be examined with a numerical pain rating scale (0-100) during a shortened protocol of two sets of seven pseudo-random pin-prick stimulations.[29] To determine the presence of allodynia, two sets of three light touch stimulations with a cotton wisp, a cotton wool tip, and a standardized brush (Sense-lab) will be intermingled with these pin-prick stimulations. Pressure pain thresholds (PPT) will be evaluated with a manual algometer (Wagner Instruments, USA) and vibration detection threshold (VDT) with a Rydel Seiffer tuning fork (average of three repetitions). The wind-up ratio (WUR) will be determined as the mean numerical pain rating of three trains of 10 pin-prick stimuli divided by the mean rating of three single stimuli.

A shortened QST battery will first be conducted on the hand ipsilateral to the (most) symptomatic leg (CPT, HPT and MPT on dorsum of hand; PPT over thenar eminence) to determine the presence of widespread hyperalgesia. The full QST protocol will then be performed in the area of maximal pain in the affected leg where pervious work has shown QST changes in patients with 'sciatica'.[30]

We will use healthy control data to calculate Z-scores, where each individual parameter is related to its region-, age- and gender specific reference range. We will collect our own normative data, assisted by the provision of an existing QST dataset.[31] Using a previously published algorithm[13], patients will also be assigned one of the following somatosensory profiles 1) sensory loss 2) thermal hyperalgesia 3) mechanical hyperalgesia.

Further, we will include a conditioned pain modulation (CPM) paradigm to examine the efficacy of the descending pain modulatory system. Such dynamic QST protocols have shown most promising prognostic ability in other pain conditions.[15 16] Based on current recommendations[32], we will evaluate a sequential CPM paradigm using PPT over the thenar eminence of the dominant hand (test stimulus, average of 3 repetitions) and cold-water immersion of the non-dominant hand to the level of the wrist (conditioning stimulus). This combination has provided the most reliable and large magnitude CPM effects.[33] The water bath will be standardized to 4°C ± 2°C by adding ice. Patients are asked to report

For peer review only

the intensity of pain experienced by cold water immersion from 0 (no pain) to 100 (worst pain imaginable). Once the pain reaches the cut-off of >40/100, or after a maximum of two minutes if this cut-off is not reached,[32 34] the participants will be asked to remove the hand from the water bath. The test stimulus will be repeated immediately thereafter. Cold water immersion is the most used CPM conditioning stimulus, is easy to implement and seems to be the most effective CPM paradigm.[35 36] PPT measurements are convenient, quickly measured and frequently used as a test stimulus.[37] A good to excellent intra-session reliability for CPM assessment with PPTs has been reported.[36 38]

3) Psychosocial profiles

There is a large body of evidence supporting the role of psychosocial factors in the persistence of pain and disability.[39 40] Therefore, we will assess psychosocial factors to examine their prognostic value in sciatica. The selection of specific measures of psychosocial factors drew upon existing evidence for their predictive utility in the context of other pain conditions, their theoretical relevance, and their psychometric properties including content validity.[41] We will have a two-level approach to assessment that includes general or "transdiagnostic" psychosocial factors and condition/sciatica-specific factors (Table 1). The transdiagnostic factors include symptoms of depression and general anxiety, sleep disturbance, and fatigue (all measured with their respective PROMIS SF8a tools[42]), trauma history, pain-related worry ("Pain Catastrophizing Scale")[43] and personality (Ten Item Personality Inventory[44]). In addition to transdiagnostic psychosocial risk factors, we have included several measures of potential protective factors (ie, optimism, State Optimism Measure[45]; social support, PROMIS SF4a instrumental and emotional Support; and social role participation, PROMIS SF8a) to provide a more holistic assessment. To assess cognitions specific to the context of sciatica, we developed a novel item set that was primarily adapted from the revised Illness Perception Questionnaire (Appendix 2).[46] Patient partners provided extensive feedback to develop and refine the sciatica-specific adaptation of these items. We have also included a measure of stigma[47] in relation to sciatica.

4) Blood inflammatory markers

We will sample blood by cubital venepuncture into BD Vacutainer SST and serum clot activator tubes (gold and red cap, BD, Wokingham United Kingdom). The time of last meal will be recorded. Thirty minutes after venepuncture, the blood will be centrifuged at 1.3g for 10 minutes at 4°C (gold cap for protein analysis) and at room temperature (red cap tubes for metabolomics). The serum fraction will be immediately aliquoted and stored at -80°C for batch processing.

We will use complimentary protein/metabolomics analysis to evaluate serum inflammatory markers related to inflammation and neuropathic pain. Protein analysis will utilise a custom-made electro-chemiluminescent multiplex biomarkers assays (MSD) available at Oxford. These plates contain 17

For peer review only

cytokines/chemokines including candidates of interest derived in our previous work (e.g., IL-4, IL-9, IL-6).[48] Patient samples will be run in duplicate and normalised to standard curves.

Metabolomic analyses will be carried out using a state-of-the-art, high-field 700 MHz NMR spectrometer equipped with TCI cryoprobe (Department of Chemistry, University of Oxford), as previously described.[49] Quality control samples will be randomly spread throughout the run for standardisation and internal reference standards will allow absolute concentrations of inflammatory markers (N-acetylated glycoprotein species, serum lipoproteins,) along with energy and TCA-cycle metabolites to be determined.

For peer review only

Additional phenotypic data

Demographics and medical information

We will also collect basic demographic data (e.g., age, gender, ethnicity, profession, working status, perception of household income, years of school attendance) and medical information (e.g., most affected side, previous history of back pain or sciatica, number of previous episodes, duration of current episode, family history of pain, current and past medical history including current and past medications and their effectiveness, trialled treatments, results of previous imaging, smoking and alcohol intake, Appendix 3).

Clinical Examination

We will also perform a clinical examination (Appendix 4). We will document height, weight and hip/waist circumference. We will record findings from a bedside neurological screening examination of the lower limbs. This includes myotomal testing from lumbar levels L2-S1, patellar and achilles tendon reflexes, as well as mapping of sensory loss to light touch and pin prick on body charts. We will check for upper motor neurone signs (exclusion criteria) using Hoffmann's test, Babinski, inverted supinator sign and observation of tandem walk.[50] Patients will go through a warning sign checklist for suspected cauda equina syndrome (exclusion criteria).[51]

We will perform the straight leg raise and slump test as well as femoral slump if indicated (e.g., presentation suggesting upper lumbar involvement).[52] These tests for nerve mechanosensitivity will be deemed positive if they 1) reproduce at least partially the patients' symptoms and 2) if structural differentiation through either foot dorsiflexion or cervical flexion changes the symptoms. [53] We will further record the presence of lumbar shifts, active range of motion restrictions in lower back and hip including whether these movements provoke back or leg symptoms. Pain provocation upon posterior anterior intervertebral movement palpation of the lumbar segments L1-L5 will be recorded (Grade IV unless pain provocation occurs earlier).

At the end of the baseline appointment, the assessor will rate the certainty of neuropathic leg pain as unlikely, possible, probable or definite according to the updated neuropathic pain grading system.[54] They will also assign patients to one or several of the following subgroups described elsewhere[55]: radiculopathy (true neurological deficit), radicular pain, neural mechanosensitivity or somatic referred pain.

Self-reported questionnaires

We will also collect the following additional questionnaires to describe our patient population: ODI[56] (separate questionnaires for back and leg), Keele Start Back tool, [57] EQ-5D,[58] and a monthly pain diary (Appendix 3).

Magnetic resonance neurography (MRN)

We will perform MRN in a subset of n=100 patients with sciatica and n=44 healthy matched controls to identify moderate effects[23] (d=0.52, alpha=0.05, 80% power). Eligible patients (e.g., MRI safety) will be consecutively recruited for scanning until numbers are reached.

We will perform advanced MRN optimised to visualise lumbar nerve root macro- and microstructure at 3 Tesla using a dedicated 18-channel phased array spine coil (Siemens, UK). The protocol includes multi-shell (b=700 and 1500 s/mm2) DTI scans, high resolution anatomic scans with optimised T1 and T2 weighted contrasts, and a T2 mapping scan (Appendix 5). The data analysis will be performed using FSL tools including TOPUP[59 60] and EDDY [61-63] for the correction of images' distortions and subject movements, DTIFIT[64] for the fitting of diffusion tensor model, and FLIRT[65 66] for the registration of diffusion metrics and anatomic images. Measures including fractional anisotropy, mean/axial/radial diffusivity and T2 maps will be obtained within regions of interest in lumbar nerve roots (affected and unaffected sides) and averaged over multiple slices as we have optimised before.[67]

Cohort harmonisation

The FORECAST cohort is harmonised with the Advanced Pain Discovery Platform funded PAINSTORM consortium, and therefore includes additional measures that will allow data integration (e.g., blood collection for genetic analyses, skin biopsies in the maximal pain area, DN4,[68] Michigan Neuropathy Screening Instrument,[69] Chronic pain grade,[70] Brief pain inventory[71] (pain intensity items), a section where patients can tell us more about their pain and circumstances in their own words including how they would describe their pain to their friends/family or work colleagues, as well as their feelings about their financial situation and its impact on their situation.

For peer review only

Data analysis plan

Statistical methods will follow STROBE guidelines[17] and the TRIPOD statement for transparent reporting of a multivariable prediction model for individual prognosis or diagnosis.[18]

Participants' baseline characteristics (e.g., demographics, disability using (ODI), medical co-morbidities) and their clinical course (primary and secondary outcomes, ODI) will be described for short (3 months) and long-term time-points (12 months).

To identify and characterise mechanism-based subgroups in patients with acute/subacute sciatica and use distance-based clustering algorithms efficiently we first need to address the high dimensionality – modest sample size of the dataset. Thus, we will first carry out a Principal Component Analysis (PCA) to summarise and reduce the dimensionality of the dataset while preserving as much variability as possible. Then we will use algorithmic centroid (k-means) and hierarchical clustering based on the Euclidean distance between principal dimensions to identify sub-groups of patients sharing high phenotypic similarities. The optimal number of clusters will be determined using the gap statistic and the elbow of the within/between clusters variance plot. Consequently, we will perform hypothesis testing to assess group differences on the original variables between participants assigned to different clusters. All omnibus tests will be followed-up by the appropriate post-hoc test.

To investigate factors that predict pain persistence in people with sciatica we will use variable selection techniques followed by predictive modelling. First, we will perform filtering of the original variables by calculating the univariate associations (coefficients, 95% CI, p-values) between variables and the outcome and between each other. We will select a subset of uncorrelated variables that are associated with the outcome and use them as input features in machine learning algorithms for high dimensional, small datasets that will allow us to identify the most powerful predictors and assess model selection/predictive accuracy. During pre-processing, missing data will be examined, the mechanism of missingness will be inferred using hypothesis testing and visually assessed using a matrix of boxplots for all pairs of variables and the outcome, and if appropriate multiple imputation by chained equations will be used. Drawing from machine learning techniques for high dimensional small datasets we will use re-sampling and validation in the form of repeated cross-validation to perform a complete variable profiling to identify the most powerful predictors. Multivariate Adaptive Regression Splines (MARS) with built-in feature selection and Decision Tree models known to work well on low sample sizes will be trained to predict the 3-month and 1-year outcome. Model performance will be estimated using 5-times repeated 10-fold cross-validation and compared to models trained on surrogate data.[72] The latter benchmarking technique is appropriate for small datasets, where holding out a subset of data before the analysis to be used as a pseudo-independent test set is impossible. Instead, an artificial – surrogate

For peer review only

dataset, preserving the descriptive statistics but not any of the potentially real associations between the variables and the outcome of the original dataset, will be created and the performance of models trained on the actual and surrogate dataset will be compared. Models' predictive performance will be reported alongside variable importance rankings. Model selection will be done to maximise the Mathews Correlation Coefficient for dichotomised outcomes and to minimise the Root Mean Square Error (RMSE) for continuous outcomes during cross-validation. Scalar metric estimations of predictive performance including accuracy (binomial test p-value against the majority class prevalence), balanced accuracy and the area under the precision/recall curve will be reported alongside their 95% CI. Predictor importance will be assessed using model specific techniques, i.e., the reduction in performance estimated by cross-validation when each predictor is removed for MARS and node impurity for tree-based methods. Variables' influence on the predicted outcome both at the global and individual level will be quantified by the Partial Dependence Plots and Individual Conditional Expectation[73] respectively. These will show the average marginal effect on the prediction given a certain value of a predictor variable and provide model interpretability.

Sample size estimation

QST sensory profiles: Published sample size guidelines for QST clustering in peripheral nerve injury[74] suggest that for strong effects (effect size = 0.7) a sample size of <180 patients will produce a subpopulation with thermal and mechanical hyperalgesia large enough to conduct a study with 80% power, at an alpha 0.05. To calculate QST z-scores, at least 8 controls are required for each area and age decade.[75] Our feasibility study included patients of 7 age decades with 3 main pain areas. We will therefore need n=168 controls.

k-means and hierarchical clustering after PCA: Using the 2 first principal dimensions for 3 variable domains (self-reported profiling, QST, inflammatory markers) we will need 2^6=64 patients to perform k-means clustering with adequate power.[76]

Algorithmic cluster analysis: assuming k=4 clusters, we will be able to identify moderate effects (effect size = 0.25) with an one-way ANOVA between 4 groups at an alpha level of 0.05, 80% power.

Predictor profiling: we will use robust algorithms that include feature selection, and we will assess model performance using methods developed for small datasets and robust metrics. As this part is an exploratory analysis that could shape future hypotheses and validation studies, our sample size is adequate. Given the anticipated sample size ratios with chronic (180*30%=54) and resolved sciatica (180*70%=126) and accounting for 15% attrition (see feasibility study), we will be able to identify moderate effects (effect size = 0.5) using a two-tailed Wilcoxon-Mann-Whitney test (power 81%, alpha 0.05).

For peer review only

Ethics and dissemination

The FORECAST study has received ethical approval (South Central Oxford C, 18/SC/0263). All participants will provide informed written consent before participating in the study.

The dissemination strategy will be strongly guided by our PPIE activities (see below). This will be based on co-productions between patient partners and academics and will involve publication of findings in scientific journals, presentations at conferences, media pieces (mainstream and social media) as well as communication through charity partners.

Data will be made publicly available on the ALLEVIATE data hub (https://alleviate.ac.uk) and remaining bio-samples will be on-boarded to the Imperial Biobank. The data and samples will continue to be linked and will be available for future studies.

Patient and Public Involvement and Engagement (PPIE) and Dissemination of Findings

The FORECAST team consists of equal partners including patient partners, clinicians and researchers. Our aims have been shaped by the needs of people living with sciatica to ensure we address unmet needs. The PPIE plans will be shaped by the following members of FORECAST: 1) Inclusion of two patient partners as co-investigators (CR, CP). They will contribute as equal partners on the investigator team. 2) PPIE lead with extensive experience in involving patients' voices in research (KRM). 3) diverse patient advisory group (PAG) consisting of six individuals with a lived experience of sciatica. Our patient partners and PAG provided early input to the original grant application and identification of key research activities within the project, particularly around including the feasibility work (e.g., acceptability of testing and study procedures), study design (e.g., selection of primary outcome measure) and strongly informed the writing of our funding application (e.g., lay summary). We will continue to work closely with people with lived experience of sciatica as we undertake this study, and our PPIE strategy will continue to be implemented throughout the lifetime of FORECAST. We will seek the perspective and guidance of our patient partners and PAG members on matters including, but not limited to participant recruitment and retention; barriers/facilitators of participation among seldom heard populations; data analysis and sensemaking of findings, organisation and co-production of workshops, dissemination materials, and public engagement activities. This will ensure that the patient perspective has been considered at all stages throughout the project.

We will also work closely with our patient partners and advisors on engagement and dissemination activities. This may include, but is not limited to, co-producing lay summaries, website content, infographics, animated videos, and podcasts, as well as engagement activities to bring the project into a public sphere. We plan to work closely with the PAINSTORM research team and patient partners, as well as other national and international pain and sciatica groups to promote the study and its

For peer review only

subsequent findings. This would allow us to reflect on the way the conclusions are presented and identify any gaps which might lead to further research in the topic area. We also plan to hold conversations with our patient partners and PAG regarding planning and undertaking academic dissemination activities (e.g., engagement with policy stakeholders, conference abstracts/presentations, manuscript preparation/publication). All individuals who contribute to this PPI advisory group will receive payment in accordance with current INVOLVE guidelines.

For peer review only

Acknowledgement

The authors wish to thank all participants of the feasibility study who have helped shape the protocol of the FORECAST study. The input of Prof Irene Tracey in the design of the study is also gratefully acknowledged.

Author contributions:

ABS has conceived the FORECAST study and acquired funding with the support of all FORECAST collaborators. LR is coordinating the FORECAST study and SK is supporting data collection. LH coordinated the feasibility study. FP assisted in the design of the blood marker analysis and will perform all metabolomics data acquisition, processing, and analyses. WS and GC led on the psychosocial profiling. MT, SC, DN, and SA contributed to the development of the MRI sequences and will perform MRI-related analyses. CP, CR and KM are responsible for the PPIE. BT has provided input into the QST assessment and will provide normative QST datasets. MB will provide the SYP body diagram software and run the pain drawing analyses. JF will provide clinical oversight. GB performed sample size analyses and will be responsible for overall data integration and analysis. All authors have contributed to the study design and write up of the protocol and approved the final version.

Funding statement:

This project is funded by UKRI and Versus Arthritis as part of the UKRI Strategic Priorities Fund (SPF) Advanced Pain Discovery Platform (APDP), a co-funded initiative by UKRI (MRC, BBSRC, ESRC), Versus Arthritis, the Medical Research Foundation and Eli Lilly and Company Ltd (Grant MR/W027003/1).

ABS is supported by a Wellcome Trust Clinical Career Development Fellowship (222101/Z/20/Z).

BT received funding to collect normative QST data from the Government of Western Australia, Department of Health and the Raine Medical Research Foundation, the Sir Charles Gairdner Hospital and Osborne Park Health Care Group Research Advisory Committee Grant (RAC 2016-17/015) and the Charlies Foundation for Research, Arthritis Australia (The Eventide Homes Grant) and School of Physiotherapy and Exercise Science, Curtin University.

WS is partly funded through the National Institute for Health and Care Research (NIHR) Biomedical Research Centre at the South London and Maudsley NHS Foundation Trust and King's College London.

FP is funded by a Dorothy Hodgkin Career Development Fellowship in Chemistry in association with Somerville College.

GB is supported by the Wellcome Trust, grant reference 223149/Z/21/Z and Diabetes UK, grant reference 19/0005984.

For peer review only

GC and KRM are partly funded by UKRI and Versus Arthritis as part of the Advanced Pain Discovery Platform (APDP) PAINSTORM (MR/W002388/1).

LH was supported by a preparatory research fellowship from the NIHR Biomedical Research Centre Oxford, based at Oxford University Hospital NHS Foundation Trust, Oxford. The research is supported by the National Institute for Health Research (NIHR) Oxford Health Biomedical Research Centre (BRC). The views expressed are those of the authors and not necessarily those of the NHS, the NIHR or the Department of Health and Social Care

Data statement: Data will be made publicly available on the ALLEVIATE data hub (https://alleviate.ac.uk) and remaining bio-samples will be on-boarded to the Imperial Biobank. The data and samples will continue to be linked and will be available for future studies.

Competing interest statement: The authors declare no competing interests.

For peer review only

References

1. Wu A, March L, Zheng X, et al. Global low back pain prevalence and years lived with disability from 1990 to 2017: estimates from the Global Burden of Disease Study 2017. *Ann Transl Med* 2020;8(6):299. doi: 10.21037/atm.2020.02.175 [published Online First: 2020/05/02]
2. Kleinman N, Patel AA, Benson C, et al. Economic burden of back and neck pain: effect of a neuropathic component. *Population health management* 2014;17(4):224-32. doi: 10.1089/pop.2013.0071
3. Centre NG. NICE guideline NG59: Low back pain and sciatica in over 16s: assessment and management. In: UK NIfHaCE, ed., 2016.
4. Pinto RZ, Maher CG, Ferreira ML, et al. Drugs for relief of pain in patients with sciatica: systematic review and meta-analysis. *BMJ* 2012;344:e497. doi: 10.1136/bmj.e497 [published Online First: 2012/02/15]
5. Dove L, Jones G, Kelsey LA, et al. How effective are physiotherapy interventions in treating people with sciatica? A systematic review and meta-analysis. *Eur Spine J* 2022 doi: 10.1007/s00586-022-07356-y [published Online First: 20221229]
6. Haugen AJ, Brox JI, Grovle L, et al. Prognostic factors for non-success in patients with sciatica and disc herniation. *BMC Musculoskelet Disord* 2012;13:183. doi: 10.1186/1471-2474-13-183
7. Iversen T, Solberg TK, Wilsgaard T, et al. Outcome prediction in chronic unilateral lumbar radiculopathy: prospective cohort study. *BMC Musculoskelet Disord* 2015;16:17. doi: 10.1186/s12891-015-0474-9 [published Online First: 2015/04/19]
8. Konstantinou K, Dunn KM, Ogollah R, et al. Prognosis of sciatica and back-related leg pain in primary care: the ATLAS cohort. *Spine J* 2018;18(6):1030-40. doi: 10.1016/j.spinee.2017.10.071 [published Online First: 2017/11/28]
9. Vroomen PC, de Krom MC, Slofstra PD, et al. Conservative treatment of sciatica: a systematic review. *J Spinal Disord* 2000;13(6):463-9. [published Online First: 2001/01/02]
10. Weber H, Holme I, Amlie E. The natural course of acute sciatica with nerve root symptoms in a double-blind placebo-controlled trial evaluating the effect of piroxicam. *Spine (Phila Pa 1976)* 1993;18(11):1433-8. [published Online First: 1993/09/01]
11. Ashworth J, Konstantinou K, Dunn KM. Prognostic factors in non-surgically treated sciatica: a systematic review. *BMC Musculoskelet Disord* 2011;12:208. doi: 10.1186/1471-2474-12-208 [published Online First: 2011/09/29]
12. Verwoerd AJ, Luijsterburg PA, Lin CW, et al. Systematic review of prognostic factors predicting outcome in non-surgically treated patients with sciatica. *Eur J Pain* 2013;17(8):1126-37. doi: 10.1002/j.1532-2149.2013.00301.x
13. Baron R, Maier C, Attal N, et al. Peripheral neuropathic pain: a mechanism-related organizing principle based on sensory profiles. *Pain* 2017;158(2):261-72. doi: 10.1097/j.pain.0000000000000753
14. Baron R, Wasner G, Binder A. Chronic pain: genes, plasticity, and phenotypes. *Lancet Neurol* 2012;11(1):19-21. doi: 10.1016/S1474-4422(11)70281-2 [published Online First: 2011/12/17]
15. Petersen KK, Vaegter HB, Stubhaug A, et al. The predictive value of quantitative sensory testing: a systematic review on chronic postoperative pain and the analgesic effect of pharmacological therapies in patients with chronic pain. *Pain* 2021;162(1):31-44. doi: 10.1097/j.pain.0000000000002019 [published Online First: 2020/07/24]
16. Georgopoulos V, Akin-Akinyosoye K, Zhang W, et al. Quantitative sensory testing and predicting outcomes for musculoskeletal pain, disability, and negative affect: a systematic review and meta-analysis. *Pain* 2019;160(9):1920-32. doi: 10.1097/j.pain.0000000000001590 [published Online First: 2019/05/03]
17. von Elm E, Altman DG, Egger M, et al. The Strengthening the Reporting of Observational Studies in Epidemiology (STROBE) statement: guidelines for reporting observational studies. *Lancet* 2007;370(9596):1453-7. doi: 10.1016/S0140-6736(07)61602-X [published Online First: 2007/12/08]

For peer review only

18. Collins GS, Reitsma JB, Altman DG, et al. Transparent reporting of a multivariable prediction model for individual prognosis or diagnosis (TRIPOD): the TRIPOD statement. *BMJ* 2015;350:g7594. doi: 10.1136/bmj.g7594 [published Online First: 2015/01/09]
19. Stynes S, Konstantinou K, Ogollah R, et al. Clinical diagnostic model for sciatica developed in primary care patients with low back-related leg pain. *PLoS ONE* 2018;13(4):e0191852. doi: 10.1371/journal.pone.0191852 [published Online First: 2018/04/06]
20. Patrick DL, Deyo RA, Atlas SJ, et al. Assessing health-related quality of life in patients with sciatica. *Spine (Phila Pa 1976)* 1995;20(17):1899-908; discussion 909. [published Online First: 1995/09/01]
21. Haugen AJ, Grovle L, Brox JI, et al. Estimates of success in patients with sciatica due to lumbar disc herniation depend upon outcome measure. *Eur Spine J* 2011;20(10):1669-75. doi: 10.1007/s00586-011-1809-3 [published Online First: 2011/04/26]
22. Fairbank J. Oswestry Disability Index: ePROVIDE; 2022 [Available from: https://eprovide.mapi-trust.org/instruments/oswestry-disability-index2022.
23. Jaeschke R, Singer J, Guyatt GH. Measurement of health status. Ascertaining the minimal clinically important difference. *Control Clin Trials* 1989;10(4):407-15. [published Online First: 1989/12/01]
24. Mahn F, Hullemann P, Gockel U, et al. Sensory symptom profiles and co-morbidities in painful radiculopathy. *PLoS ONE* 2011;6(5):e18018. doi: 10.1371/journal.pone.0018018
25. Willett MJ, Siebertz M, Petzke F, et al. The Extent of Pain Is Associated With Signs of Central Sensitization in Patients With Hip Osteoarthritis. *Pain Pract* 2020;20(3):277-88. doi: 10.1111/papr.12851 [published Online First: 2019/10/31]
26. Lluch Girbes E, Duenas L, Barbero M, et al. Expanded Distribution of Pain as a Sign of Central Sensitization in Individuals With Symptomatic Knee Osteoarthritis. *Phys Ther* 2016;96(8):1196-207. doi: 10.2522/ptj.20150492 [published Online First: 2016/03/05]
27. Evans DW, Rushton A, Middlebrook N, et al. Estimating Risk of Chronic Pain and Disability Following Musculoskeletal Trauma in the United Kingdom. *JAMA Netw Open* 2022;5(8):e2228870. doi: 10.1001/jamanetworkopen.2022.28870 [published Online First: 2022/08/27]
28. Alter BJ, Anderson NP, Gillman AG, et al. Hierarchical clustering by patient-reported pain distribution alone identifies distinct chronic pain subgroups differing by pain intensity, quality, and clinical outcomes. *PLoS ONE* 2021;16(8):e0254862. doi: 10.1371/journal.pone.0254862 [published Online First: 2021/08/05]
29. Pascal MMV, Themistocleous AC, Baron R, et al. DOLORisk: study protocol for a multi-centre observational study to understand the risk factors and determinants of neuropathic pain. *Wellcome Open Res* 2018;3:63. doi: 10.12688/wellcomeopenres.14576.2 [published Online First: 2019/02/16]
30. Tampin B, Slater H, Jacques A, et al. Association of quantitative sensory testing parameters with clinical outcome in patients with lumbar radiculopathy undergoing microdiscectomy. *Eur J Pain* 2020;24(7):1377-92. doi: 10.1002/ejp.1586 [published Online First: 2020/05/10]
31. Zhu GC, Bottger K, Slater H, et al. Concurrent validity of a low-cost and time-efficient clinical sensory test battery to evaluate somatosensory dysfunction. *Eur J Pain* 2019;23(10):1826-38. doi: 10.1002/ejp.1456 [published Online First: 2019/07/22]
32. Yarnitsky D, Bouhassira D, Drewes AM, et al. Recommendations on practice of conditioned pain modulation (CPM) testing. *Eur J Pain* 2015;19(6):805-6. doi: 10.1002/ejp.605 [published Online First: 2014/10/21]
33. Imai Y, Petersen KK, Morch CD, et al. Comparing test-retest reliability and magnitude of conditioned pain modulation using different combinations of test and conditioning stimuli. *Somatosens Mot Res* 2016;33(3-4):169-77. doi: 10.1080/08990220.2016.1229178 [published Online First: 2016/09/22]
34. Nir RR, Granovsky Y, Yarnitsky D, et al. A psychophysical study of endogenous analgesia: the role of the conditioning pain in the induction and magnitude of conditioned pain modulation. *Eur J Pain* 2011;15(5):491-7. doi: 10.1016/j.ejpain.2010.10.001 [published Online First: 2010/11/03]

35. Aparecida da Silva V, Galhardoni R, Teixeira MJ, et al. Not just a matter of pain intensity: Effects of three different conditioning stimuli on conditioned pain modulation effects. *Neurophysiol Clin* 2018;48(5):287-93. doi: 10.1016/j.neucli.2018.06.078 [published Online First: 2018/06/30]
36. Kennedy DL, Kemp HI, Ridout D, et al. Reliability of conditioned pain modulation: a systematic review. *Pain* 2016;157(11):2410-19. doi: 10.1097/j.pain.0000000000000689 [published Online First: 2016/10/19]
37. Pud D, Granovsky Y, Yarnitsky D. The methodology of experimentally induced diffuse noxious inhibitory control (DNIC)-like effect in humans. *Pain* 2009;144(1-2):16-9. doi: 10.1016/j.pain.2009.02.015 [published Online First: 2009/04/11]
38. Lewis GN, Rice DA, McNair PJ. Conditioned pain modulation in populations with chronic pain: a systematic review and meta-analysis. *J Pain* 2012;13(10):936-44. doi: 10.1016/j.jpain.2012.07.005 [published Online First: 2012/09/18]
39. Giusti EM, Lacerenza M, Manzoni GM, et al. Psychological and psychosocial predictors of chronic postsurgical pain: a systematic review and meta-analysis. *Pain* 2021;162(1):10-30. doi: 10.1097/j.pain.0000000000001999 [published Online First: 2020/07/23]
40. Hruschak V, Cochran G. Psychosocial predictors in the transition from acute to chronic pain: a systematic review. *Psychol Health Med* 2018;23(10):1151-67. doi: 10.1080/13548506.2018.1446097 [published Online First: 2018/03/02]
41. Crombez G, De Paepe AL, Veirman E, et al. Let's talk about pain catastrophizing measures: an item content analysis. *Peerj* 2020;8 doi: ARTN e8643
10.7717/peerj.8643
42. Cella D, Yount S, Rothrock N, et al. The Patient-Reported Outcomes Measurement Information System (PROMIS): progress of an NIH Roadmap cooperative group during its first two years. *Med Care* 2007;45(5 Suppl 1):S3-S11. doi: 10.1097/01.mlr.0000258615.42478.55
43. Sullivan MJL, Bishop SR, Pivik J. The Pain Catastrophizing Scale: Development and Validation. *Psychol Assess* 1995;7:4.
44. Gosling SD, Rentfrow PJ, Swann WB. A very brief measure of the Big-Five personality domains. *Journal of Research in Personality* 2003;37(6):504-28. doi: 10.1016/S0092-6566(03)00046-1
45. Millstein RA, Chung WJ, Hoeppner BB, et al. Development of the State Optimism Measure. *Gen Hosp Psychiatry* 2019;58:83-93. doi: 10.1016/j.genhosppsych.2019.04.002 [published Online First: 2019/04/27]
46. Moss-Morris R, Weinman J, Petrie KJ, et al. The revised Illness Perception Questionnaire (IPQ-R). *Psychology & Health* 2002;17(1):1-16. doi: 10.1080/08870440290001494
47. Molina Y, Choi SW, Cella D, et al. The Stigma Scale for Chronic Illnesses 8-Item Version (SSCI-8): Development, Validation and Use Across Neurological Conditions. *International Journal of Behavioral Medicine* 2013;20(3):450-60. doi: 10.1007/s12529-012-9243-4
48. Sandy-Hindmarch O, Bennett DL, Wiberg A, et al. Systemic inflammatory markers in neuropathic pain, nerve injury and recovery. *Pain* 2021 doi: 10.1097/j.pain.0000000000002386 [published Online First: 2021/07/06]
49. Probert F, Yeo T, Zhou Y, et al. Integrative biochemical, proteomics and metabolomics cerebrospinal fluid biomarkers predict clinical conversion to multiple sclerosis. *Brain Commun* 2021;3(2):fcab084. doi: 10.1093/braincomms/fcab084 [published Online First: 2021/05/18]
50. Cook C, Brown C, Isaacs R, et al. Clustered clinical findings for diagnosis of cervical spine myelopathy. *J Man Manip Ther* 2010;18(4):175-80. doi: 10.1179/106698110X12804993427045 [published Online First: 2011/12/02]
51. Greenhalgh S, Finucane L, Mercer C, et al. Assessment and management of cauda equina syndrome. *Musculoskelet Sci Pract* 2018;37:69-74. doi: 10.1016/j.msksp.2018.06.002 [published Online First: 2018/06/25]
52. Butler DS. The sensitive nervous system. Adelaide: NOIgroup publications 2000.
53. Nee RJ, Jull GA, Vicenzino B, et al. The validity of upper-limb neurodynamic tests for detecting peripheral neuropathic pain. *J Orthop Sports Phys Ther* 2012;42(5):413-24. doi: 10.2519/jospt.2012.3988 [published Online First: 2012/03/10]

For peer review only

54. Finnerup NB, Haroutounian S, Kamerman P, et al. Neuropathic pain: an updated grading system for research and clinical practice. *Pain* 2016;157(8):1599-606. doi: 10.1097/j.pain.0000000000000492
55. Schmid AB, Tampin B. Spinally Referred Back and Leg Pain. In: Spine ISftSotL, ed. Lumbar Spine Online Textbook. http://www.wheelessonline.com/ISSLS/section-10-chapter-10-spinally-referred-back-and-leg-pain/2018.
56. Fairbank JC, Pynsent PB. The Oswestry Disability Index. *Spine (Phila Pa 1976)* 2000;25(22):2940-52; discussion 52.
57. Hill JC, Dunn KM, Main CJ, et al. Subgrouping low back pain: a comparison of the STarT Back Tool with the Orebro Musculoskeletal Pain Screening Questionnaire. *Eur J Pain* 2010;14(1):83-9. doi: 10.1016/j.ejpain.2009.01.003 [published Online First: 2009/02/19]
58. Group TE. EuroQol-a new facility for the measurement of health-related quality of life. *Health Policy* 1990;16(3):199-208.
59. Andersson JL, Skare S, Ashburner J. How to correct susceptibility distortions in spin-echo echo-planar images: application to diffusion tensor imaging. *Neuroimage* 2003;20(2):870-88. doi: 10.1016/S1053-8119(03)00336-7
60. Smith SM, Jenkinson M, Woolrich MW, et al. Advances in functional and structural MR image analysis and implementation as FSL. *Neuroimage* 2004;23 Suppl 1:S208-19. doi: 10.1016/j.neuroimage.2004.07.051
61. Andersson JLR, Sotiropoulos SN. An integrated approach to correction for off-resonance effects and subject movement in diffusion MR imaging. *Neuroimage* 2016;125:1063-78. doi: 10.1016/j.neuroimage.2015.10.019 [published Online First: 20151020]
62. Andersson JLR, Graham MS, Drobnjak I, et al. Towards a comprehensive framework for movement and distortion correction of diffusion MR images: Within volume movement. *Neuroimage* 2017;152:450-66. doi: 10.1016/j.neuroimage.2017.02.085 [published Online First: 20170308]
63. Andersson JLR, Graham MS, Drobnjak I, et al. Susceptibility-induced distortion that varies due to motion: Correction in diffusion MR without acquiring additional data. *Neuroimage* 2018;171:277-95. doi: 10.1016/j.neuroimage.2017.12.040 [published Online First: 20171224]
64. Sotiropoulos SN, Hernandez-Fernandez M, Vu AT, et al. Fusion in diffusion MRI for improved fibre orientation estimation: An application to the 3T and 7T data of the Human Connectome Project. *Neuroimage* 2016;134:396-409. doi: 10.1016/j.neuroimage.2016.04.014 [published Online First: 20160409]
65. Jenkinson M, Smith S. A global optimisation method for robust affine registration of brain images. *Med Image Anal* 2001;5(2):143-56.
66. Jenkinson M, Bannister P, Brady M, et al. Improved optimization for the robust and accurate linear registration and motion correction of brain images. *Neuroimage* 2002;17(2):825-41.
67. Schmid AB, Campbell J, Hurley SA, et al. Feasibility of Diffusion Tensor and Morphologic Imaging of Peripheral Nerves at Ultra-High Field Strength. *Invest Radiol* 2018;53(12):705-13. doi: 10.1097/RLI.0000000000000492 [published Online First: 2018/07/07]
68. Bouhassira D, Attal N, Alchaar H, et al. Comparison of pain syndromes associated with nervous or somatic lesions and development of a new neuropathic pain diagnostic questionnaire (DN4). *Pain* 2005;114(1-2):29-36. doi: 10.1016/j.pain.2004.12.010 [published Online First: 2005/03/01]
69. Feldman EL, Stevens MJ, Thomas PK, et al. A practical two-step quantitative clinical and electrophysiological assessment for the diagnosis and staging of diabetic neuropathy. *Diabetes Care* 1994;17(11):1281-9.
70. Dixon D, Pollard B, Johnston M. What does the chronic pain grade questionnaire measure? *Pain* 2007;130(3):249-53. doi: 10.1016/j.pain.2006.12.004
71. Cleeland CS, Ryan KM. Pain assessment: global use of the Brief Pain Inventory. *Ann Acad Med Singap* 1994;23(2):129-38. [published Online First: 1994/03/01]
72. Hirata Y, Katori Y, Shimokawa H, et al. Testing a neural coding hypothesis using surrogate data. *J Neurosci Methods* 2008;172(2):312-22. doi: 10.1016/j.jneumeth.2008.05.004 [published Online First: 2008/06/21]

For peer review only

73. Goldstein A, Kapelner A, Bleich J, et al. Peeking Inside the Black Box: Visualizing Statistical Learning with PLots of Individual Conditional Expectation. *Journal of Computational and Graphical Statistics* 2015;24(1):44-65.
74. Vollert J, Maier C, Attal N, et al. Stratifying patients with peripheral neuropathic pain based on sensory profiles: algorithm and sample size recommendations. *Pain* 2017;158(8):1446-55. doi: 10.1097/j.pain.0000000000000935 [published Online First: 2017/06/09]
75. Blankenburg M, Boekens H, Hechler T, et al. Reference values for quantitative sensory testing in children and adolescents: developmental and gender differences of somatosensory perception. *Pain* 2010;149(1):76-88. doi: 10.1016/j.pain.2010.01.011
76. Hastie T, Tibshirani R. Generalized Additive Models. *Statistical Sciences* 1986;1:297-318.
77. Freynhagen R, Baron R, Gockel U, et al. painDETECT: a new screening questionnaire to identify neuropathic components in patients with back pain. *Curr Med Res Opin* 2006;22(10):1911-20. doi: 10.1185/030079906X132488
78. Bouhassira D, Attal N, Fermanian J, et al. Development and validation of the Neuropathic Pain Symptom Inventory. *Pain* 2004;108(3):248-57. doi: 10.1016/j.pain.2003.12.024 [published Online First: 2004/03/20]
79. Craig CL, Marshall AL, Sjostrom M, et al. International physical activity questionnaire: 12-country reliability and validity. *Med Sci Sports Exerc* 2003;35(8):1381-95. doi: 10.1249/01.MSS.0000078924.61453.FB
80. Basu S, Poole J. The Brief Illness Perception Questionnaire. *Occup Med (Lond)* 2016;66(5):419-20. doi: 10.1093/occmed/kqv203 [published Online First: 2016/06/19]

For peer review only

Figure legends:

Figure 1: Study flow diagram

MRN: magnetic resonance neurography; SBI: sciatica bothersomeness index; NPRS: numerical pain rating scale.

For peer review only

Table 1: Questionnaires

**Questionnaires**<br><br>*primary outcome<br>**secondary outcome		FORECAST patients		Healthy volunteers	PAINSTORM DATASET
		BASELINE	FOLLOW UP	BASELINE	EXTENDED
FORECAST outcomes	Sciatica Bothersomeness Index (SBI)[20]	X	X*		
	Numerical Pain Rating Scale - previous 2 weeks (worst, least, average for leg /back pain)	X	X*		
	Global Rating of Change Scale		X**		
Neuropathy/Neuropathic Pain	PainDETECT[77]	X	X		X
	Neuropathic Pain Symptom Inventory (NPSI)[78]	X	X	X	X
	DN4[68]		X		X
	Michigan Neuropathy Screening Instrument (MNSI)[69]				X
Pain location, severity	Pain location - list of sites, body chart	X	X	X	X
	Monthly pain diary	X	X		
	Chronic Pain Grade (CPG)[70]				X
	Brief Pain Inventory (BPI)[71]				X
Disability	Oswestry Disability Index (ODI 2.1a)[56] – Leg Pain	X	X**		
	Oswestry Disability Index (ODI 2.1a)[56] – Low Back Pain	X	X		
	Oswestry Disability Index (ODI 2.1a)[56] - combined leg and back pain			X	
Risk	Keele STarT Back tool[57]	X			
Lifestyle	International Physical Activity Questionnaire (IPAQ, long version)[79]	X	X	X	X

For peer review only

		FORECAST patients		Healthy volunteers	PAINSTORM DATASET
		BASELINE	FOLLOW UP	BASELINE	EXTENDED
Quality of life	EQ-5D-5L (v1.2)[58]	X	X	X	X
Psychosocial Questionnaires	PROMIS SF8a – Ability to participate in social roles and activities[42] (v1.0)	X	X	X	X
	Pain Catastrophising Scale (PCS)[43]	X	X	X	X
	PROMIS SF8-a – Depression and Anxiety[42] (v1.0)	X	X	X	X
	Adverse Childhood Events (ACEs) (none, 1, 2, >2)	X		X	X
	Prolonged hospitalisation for life threatening condition (yes/no)	X		X	X
	PROMIS SF8a – Sleep Disturbance[42] (v1.0)	X	X	X	X
	PROMIS SF8a – Fatigue[42] (v1.0)	X	X	X	X
	PROMIS SF4a-instrumental support[42] (v1.0)	X	X	X	X
	PROMIS SF4a – Emotional Support[42] (v1.0)	X	X	X	X
	Ten Item Personality Index (TIPI)[44]	X		X	X
	State Optimism Measure (SOM-7)	X	X	X	X
	Illness Perception Questionnaire (IPQ-R)[80]	X			
	Sciatica Perception Questionnaire (SPQ)	X	X		
	Stigma Scale for Chronic Illnesses (SSCI) - modified[47]	X	X		
	"in your own words" (impact on social and financial situation)				X

For peer review only

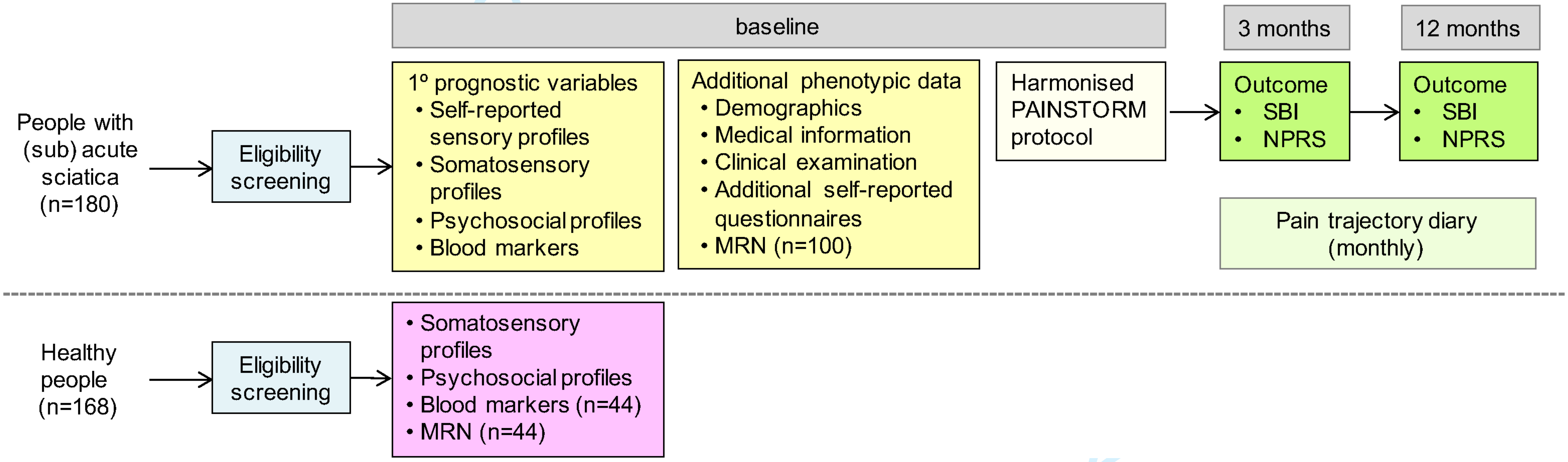

baseline
3 months
12 months
People with (sub) acute sciatica (n=180)
Eligibility screening
1º prognostic variables
• Self-reported sensory profiles
• Somatosensory profiles
• Psychosocial profiles
• Blood markers
Additional phenotypic data
• Demographics
• Medical information
• Clinical examination
• Additional self-reported questionnaires
• MRN (n=100)
Harmonised PAINSTORM protocol
Outcome
• SBI
• NPRS
Outcome
• SBI
• NPRS
Pain trajectory diary (monthly)
Healthy people (n=168)
Eligibility screening
• Somatosensory profiles
• Psychosocial profiles
• Blood markers (n=44)
• MRN (n=44)

Appendix 1: Pain trajectory Diary

ID_______________
Date_______________

Pain Trajectory Diary - FORECAST

Month:

Thank you for your continuing support of the FORECAST study. Please let us know below about your sciatica pain in the past 2 weeks.

	No pain 0	1	2	3	4	5	6	7	8	9	Worst pain imaginable 10
Sciatica leg pain											
**In the last two weeks, at its worst, how intense was your sciatica leg pain?**											
**In the last two weeks, at its least, how intense was your sciatica leg pain?**											
**In the last two weeks, on average, how intense was your sciatica leg pain?**											
Low back pain											
**In the last two weeks, on average, how intense was your back pain?**											

For peer review only

Appendix 2: Sciatica Perception Questionnaire

Your views about your sciatica (SPQ)

We are interested in your own personal views of how you currently see your sciatica.

Please indicate how much you agree or disagree with the following statements about your sciatica by ticking the appropriate box.

	Strongly disagree	Disagree	Neither agree nor disagree	Agree	Strongly agree
I expect that I am going into old age with my sciatica					
I feel that my sciatica will last for a long time					
My sciatica is likely to be permanent rather than temporary					
I expect that the effect of my sciatica on day-to-day life will worsen over time					
My sciatica comes and goes					
I do not know how my sciatica will change in the future					
My sciatica is a burden to others					
My sciatica can put me in awkward and embarrassing situations					
I have the personal strength to manage my sciatica					
I avoid specific positions and/or movements due to fear of causing pain					
I avoid specific positions and/or movements due to fear of causing damage					
There is little that I can do to improve my sciatica myself					
There is something seriously wrong with my back/leg					
The cause of my sciatica has not been investigated properly					
I am concerned about possible adverse long term consequences of the treatment for my sciatica					
There is nothing that can help my sciatica					
I do not understand what is wrong with my back/leg					
My current treatment does not make sense to me					
I worry that I am not getting the right treatment for my sciatica					
I don't know what activities I can safely do with my sciatica					
It is so unfair that I have sciatica					

For peer review only

Appendix 3: Demographic and Medical History Data

	FORECAST substudy		Healthy Volunteers	PAINSTORM DATASET
**Demographics**	BASELINE	FOLLOW UP	BASELINE	EXTENDED
Age (yrs)	X		X	X
Sex	X		X	X
Years in education	X		X	X
Working status*	X		X	X
Household income**	X		X	X
Ethnicity	X		X	X
**Medical history**				
History of sciatica (date of first episode, number of previous episodes)	X			
Duration of current sciatica episode (days)	X			
Is the leg pain worse than the back pain?	X			
Affected leg (left/right/both)	X			
Family history of chronic pain	X		X	
Details of other medical diagnoses	X		X	
Cauda equina screening questions	X			
Types of treatments received for sciatica to date	X	X		
Types of tests/ investigations undertaken for sciatica to date	X	X		
Relevant previous and current medication, including whether or not they are taken for sciatica	X	X	X	
Medications: efficacy, adherence	X	X	X	X
Tobacco and Alcohol intake	X		X	X

For peer review only

* Working status:

- ☐ In paid employment or self-employed
- ☐ Retired
- ☐ Looking after home and/or family
- ☐ Unable to work because of sickness or disability
- ☐ Unemployed
- ☐ Doing unpaid or voluntary work
- ☐ Full or part-time student
- ☐ None of the above
- ☐ Prefer not to answer

** Which of the descriptions below comes closest to how you feel about your household's income nowadays?

- ☐ Living comfortably on present income
- ☐ Coping on present income
- ☐ Finding it difficult on present income
- ☐ Finding it very difficult on present income
- ☐ Do not wish to answer
- ☐ Don't know

For peer review only

Appendix 4: Clinical Examination

**Clinical Examination (Identical for people with sciatica and healthy volunteers)**	
Height (cm)	
Weight (kg)	
Waist circumference (cm)	
Hip circumference (cm)	
Myelopathy screening cluster	Tandem gait, inverted supinator sign, Hoffman's test, Babinski reflex
Neural mechanosensitivity	straight leg raise, slump, femoral slump (where clinical picture indicates). Rated as negative or positive (at least partial symptom reproduction plus structural differentiation changes symptoms).
Lumbar spine range of motion	flexion, extension, bilateral side flexion.<br>Range recorded as full or restricted.<br>Symptom provocation recorded as: none, leg, back, leg + back.
Palpation of lumbar spine	Passive accessory intervertebral mobilisations (PAIVMS) over spinous processes L1-L5 centrally (to end of resistance if required).<br>Symptom provocation recorded as none, leg, back, leg + back

For peer review only

Appendix 5: MRI protocols

The MRN protocol includes multi-shell Diffusion Tensor Imaging (DTI) scans, high resolution coronal T1 and T2 weighted imaging, and T2 mapping scan, respectively.

- Multi-shell DTI consist of three coronal scans with three shots RESOLVE readout [56], TR/TE1/TE2=3430/46/81 ms, FOV = 256x256 mm^2, 2 mm isotropic spatial resolution, 26 slices, GRAPPA factor=2, and BW=1302Hz/Px. The first scan is acquired with b = 0 s/mm^2 and Left-Right (LR) phase encoding (PE) direction for the correction of susceptibility induced distortions. The Acquisition Time (TA) is 32 s. Each of the two other scans consist of 32 diffusion directions acquired with PE in RL direction and TA = 8min. The b-values are 700 s/mm2 and 1500 s/mm2, respectively.
- High resolution coronal T1 weighted images are acquired using a Turbo Spin Echo (TSE) sequence, TR/TE = 1050/11ms, FOV = 256x256 mm^2, 1 mm isotropic spatial resolution, 50 slices, 4 averages, GRAPPA factor = 2, Turbo Factor = 3, and TA = 6 min.
- High resolution coronal T2 weighted images are acquired using a TSE sequence, TR/TE = 4700/61ms, FOV = 256x256 mm^2, 1 mm isotropic spatial resolution, 50 slices, 4 averages, GRAPPA factor = 2, Turbo Factor = 15, with fat Saturation and TA = 9 min.
- Coronal multi-echo images are used to fit T2 maps. The acquisition parameters are: TR = 5700ms, TE = 13.8/27.6/41.4/55.2/69/82.8/96.6/110.4 ms, FOV = 256x256 mm^2, 1.3 isotropic spatial resolution, 40 slices, GRAPPA factor = 2, with fat saturation and TA = 9.5 min.

For peer review only

BMJ Open

Factors predicting the transition from acute to persistent pain in people with ‘sciatica’-the FORECAST longitudinal prognostic factor cohort study protocol

Journal:	*BMJ Open*
Manuscript ID	bmjopen-2023-072832.R1
Article Type:	Protocol
Date Submitted by the Author:	14-Mar-2023
Complete List of Authors:	Schmid, Annina B.; University of Oxford Nuffield Department of Clinical Neurosciences<br>Ridgway, Lucy; University of Oxford Nuffield Department of Clinical Neurosciences<br>Hailey, Louise; University of Oxford Nuffield Department of Clinical Neurosciences; University of Oxford Nuffield Department of Orthopaedics Rheumatology and Musculoskeletal Sciences<br>Tachrount, Mohamed; University of Oxford Nuffield Department of Clinical Neurosciences<br>Probert, Fay; University of Oxford Department of Chemistry<br>Martin, Kathryn; University of Aberdeen, Epidemiology Group, Institute of Applied Health Sciences<br>Scott, Whitney; King's College London,<br>Crombez, Geert; University of Ghent,<br>Price, Christine; University of Oxford Nuffield Department of Clinical Neurosciences<br>Robertson, Claire; University of Oxford Nuffield Department of Clinical Neurosciences<br>Koushesh, Soraya; University of Oxford Nuffield Department of Clinical Neurosciences<br>Ather, Sarim; Oxford University Hospitals NHS Foundation Trust<br>Tampin, Brigitte; Sir Charles Gairdner Hospital, Physiotherapy; Curtin School of Allied Health<br>Barbero, Marco; University of Applied Sciences and Arts of Southern Switzerland<br>Nanz, Daniel; Balgrist University Hospital, Swiss Centre for Musculoskeletal Imaging, Balgrist Campus<br>Clare, Stuart; University of Oxford Nuffield Department of Clinical Neurosciences<br>fairbank, jeremy; Nuffield Orthopaedic Centre, orthopaedic surgery<br>Baskozos, Georgios; University of Oxford, Neural Injury Group, Nuffield Department of Clinical Neuroscience, John Radcliffe Hospital
**Primary Subject Heading**:	Neurology
Secondary Subject Heading:	Anaesthesia, Diagnostics
Keywords:	Chronic Pain, Neurology < INTERNAL MEDICINE, Rehabilitation medicine < INTERNAL MEDICINE, Rheumatology < INTERNAL MEDICINE,

	Neurological injury < NEUROLOGY, Neurological pain < NEUROLOGY

Factors predicting the transition from acute to persistent pain in people with 'sciatica'-the FORECAST longitudinal prognostic factor cohort study protocol

Annina B Schmid[1,2], Lucy Ridgway[1], Louise Hailey[1,15], Mohamed Tachrount[1,2], Fay Probert[3], Kathryn R Martin[4,5], Whitney Scott[6,7], Geert Crombez[8], Christine Price[9], Claire Robertson[9], Soraya Koushesh[1], Sarim Ather[10], Brigitte Tampin[11,12,13], Marco Barbero[14], Daniel Nanz[15], Stuart Clare[1,2], Jeremy Fairbank[16], Georgios Baskozos[1]

[1] Nuffield Department of Clinical Neurosciences, University of Oxford, Oxford, UK
[2] Wellcome Centre for Integrative Neuroimaging, University of Oxford, Oxford, UK
[3] Department of Chemistry, University of Oxford, UK
[4] Academic Primary Care, Institute of Applied Health Sciences, School of Medicine, Medical Sciences and Nutrition, University of Aberdeen, UK
[5] Aberdeen Centre for Arthritis and Musculoskeletal Health, School of Medicine, Medical Sciences and Nutrition, University of Aberdeen, UK
[6] King's College London, Health Psychology Section, Institute of Psychiatry, Psychology, and Neuroscience
[7] INPUT Pain Management Unit, Guy's and St Thomas' NHS Foundation Trust, London, UK
[8] Department of Experimental-clinical and Health Psychology, Ghent University, Ghent, Belgium
[9] FORECAST Patient Partners
[10] Oxford University Hospital NHS Foundation Trust, Oxford, UK
[11] Department of Physiotherapy, Sir Charles Gairdner Hospital, Perth, Australia
[12] Curtin School of Allied Health, Faculty of Health Sciences, Curtin University, Perth, Australia
[13] Faculty of Business and Social Sciences, Hochschule Osnabrueck, University of Applied Sciences, Osnabrueck, Germany
[14] Rehabilitation Research Laboratory 2rLab, Department of Business Economics, Health and Social Care, University of Applied Sciences and Arts of Southern Switzerland, Manno, Switzerland
[15] Swiss Center for Musculoskeletal Imaging, Balgrist Campus AG and Medical Faculty, University of Zurich, Switzerland
[16] Nuffield Department of Orthopaedics, Rheumatology and Musculoskeletal Sciences, University of Oxford

Corresponding author:
Annina B Schmid
Nuffield Department of Clinical Neurosciences
John Radcliffe Hospital
West Wing Level 6

For peer review only

Headley Way
OX39DU, Oxford, UK
Phone: +44 (0)1865 223254
Email: annina.schmid@neuro-research.ch
https://www.ndcn.ox.ac.uk/team/annina-schmid

word count: 4258

For peer review only

Abstract

Introduction: Sciatica is a common condition and is associated with higher levels of pain, disability, poorer quality of life, and increased use of health resources compared to low back pain alone. Although many patients recover, a third develop persistent sciatica symptoms. It remains unclear, why some patients develop persistent sciatica as none of the traditionally considered clinical parameters (e.g., symptom severity, routine magnetic resonance imaging) are consistent prognostic factors. The FORECAST study will take a different approach by exploring mechanism-based subgroups in patients with sciatica and investigate whether a mechanism-based approach can identify factors that predict pain persistence in patients with sciatica.

Methods and analysis. We will perform a prospective longitudinal cohort study including 180 people with acute/subacute sciatica. N=168 healthy participants will provide normative data. A detailed set of variables will be assessed within 3 months after sciatica onset. This will include self-reported sensory and psychosocial profiles, quantitative sensory testing, blood inflammatory markers and advanced neuroimaging. We will determine outcome with the sciatica bothersomeness index and a numerical pain rating scale for leg pain severity at 3 and 12 months.
We will use principal component analysis followed by clustering methods to identify subgroups. Univariate associations and machine learning methods optimised for high dimensional small datasets will be used to identify the most powerful predictors and model selection/accuracy.
The results will provide crucial information about the pathophysiological drivers of sciatica symptoms and may identify prognostic factors of pain persistence.

Ethics and dissemination: The FORECAST study has received ethical approval (South Central Oxford C, 18/SC/0263). The dissemination strategy will be guided by our patient and public engagement activities and will include peer-reviewed publications, conference presentations, social media and podcasts.

Registration: ISRCTN18170726

Keywords: sciatica, radiculopathy, radicular pain, prognosis, neuropathic pain

For peer review only

Article summary

Strength and limitations

- This study has the potential to advance our understanding of the heterogeneity of pathomechanisms in people with sciatica and to identify factors that predict pain persistence.

- This dataset will include the largest deeply phenotyped 'sciatica' cohort to date.

- Harmonisation with the PAINSTORM consortium will afford integration of the FORECAST cohort into a much larger dataset of neuropathic pain.

- The large amount of data points collected for a modest cohort size will pose challenges for analyses and will require dimensionality reduction techniques

- Patient recruitment will be challenging given the time intensive phenotyping protocol. This may lead to recruitment bias.

For peer review only

Introduction

Low back pain (LBP) is associated with more disability than any other condition.[1] Up to 60% of patients with LBP also experience leg pain, which is associated with worse health outcomes. In some cases, the leg pain is caused by nerve root involvement, commonly referred to as ‘sciatica’. Whereas some patients with ‘sciatica’ have pain of predominantly nociceptive character, others develop neuropathic (nerve related) pain, which is characterised by burning pain, electric shocks or tingling. The presence of neuropathic pain in sciatica further increases suffering and disability.[2] The management of sciatica is therefore a priority. The NICE guidelines recommend a period of non-invasive treatment (e.g., medication, physiotherapy) before invasive treatment (e.g., surgery) is considered.[3] Sadly, first line management for patients with sciatica remains largely ineffective[4 5] and at least one third develops persistent pain and disability lasting a year or longer.[6-10]

It remains unclear why some patients develop persistent sciatica. Two recent systematic reviews have established that none of the traditionally considered clinical parameters (e.g. pain intensity, routine magnetic resonance imaging [MRI], mental wellbeing) are consistent prognostic factors.[11 12] Since those publications, the largest prognostic study in patients with sciatica in primary care[8] identified several factors that are weakly associated with improvement, these included shorter pain duration, belief that symptoms will not last long, myotomal weakness, overall impact of sciatica. However, at 12 months, only two factors were independently associated with outcome in the multivariable model analysis. This restricts the usefulness of predictive modelling for risk estimation of outcome for individual patients. The absence of prognostic factors hinders the early identification of patients at risk of developing persistent pain and prevents personalised treatments.

These challenges in management and risk prediction are partly attributed to a lack of understanding of the pathomechanisms at play in sciatica. Sciatica is a heterogeneous condition likely caused by differing mechanisms in individual patients,[13] which are potentially amenable to targeted treatment. In the field of neuropathic pain, mechanism-based stratification using deep phenotyping has been advocated to facilitate personalised pain management.[14] In contrast to traditionally used methods that quantify the severity of the disease with a limited battery of basic clinical measures (e.g., routine MRI scans, symptom severity basic questionnaires), a mechanism-based approach aims to stratify patients by the distinct underlying mechanisms. It has been suggested that the nature of the pathomechanisms at play in patients with pain may influence treatment outcome and prognosis.[14-16] The utility of such a mechanism-based approach in predicting pain persistence in people with sciatica remains unknown.

For peer review only

The FORECAST study will examine the value of a mechanism-based deep phenotyping approach including main domains assessing nerve function, nerve structure, inflammation and psychosocial factors.

The aims of the FORECAST study are:

1. To explore mechanism-based subgroups in patients with acute/subacute sciatica.
2. To investigate whether a mechanism-based approach can identify factors that predict pain persistence in people with sciatica.

Methods

The FORECAST study is a prospective longitudinal prognostic factor cohort study that is based on feasibility data and closely informed by patient and public involvement and engagement (PPIE) activities including feedback from our named patient partners, six-member patient advisory group, and survey results from participants of the feasibility study. The study will be performed and reported according to the guidance for observational studies (STROBE)[17] and the statement for transparent reporting of a multivariable prediction model for individual prognosis or diagnosis (TRIPOD).[18]

Participants

We will include n=180 patients with acute/subacute 'sciatica' and n=168 healthy age and gender matched participants without symptoms of sciatica/low back pain. Healthy participants are important to establish normative values for blood markers, somatosensory profiling and neuroimaging.

People aged >18 years with a clinical diagnosis of 'sciatica' will be recruited from primary care in Oxfordshire (e.g., primary care NHS providers as well as GP, Physiotherapy, Osteopathy and Chiropractor clinics) and through leaflets on public noticeboards. Sciatica symptom onset of the current episode needs to be within the past three months with a symptom free period of at least 3 months preceding the current sciatica symptoms. The inclusion criteria for patients with 'sciatica' are based on a published diagnostic model[19] which includes 5 weighted parameters (self-reported sensory changes, below knee pain, leg pain worse than back pain, neurodynamic tests, neurological deficit). A sum score >4 will be defined as sciatica, with a mean predicted probability of 83%. In addition, patients with suspected sciatica will undergo a clinical examination by a physiotherapist to further confirm the diagnosis of sciatica and rule out other diagnoses (see additional phenotypic data below).

The following exclusion criteria will apply; presence of other nerve-related disorders (e.g. diabetic neuropathy, stroke), previous lumbar spine surgery, serious spinal diseases (e.g. infection, cauda equina syndrome, metastatic lesions), chronic inflammatory disorders, other pain conditions that may confound

For peer review only

assessment (e.g., fibromyalgia), pregnancy, insufficient command of the English language to obtain consent/complete questionnaires, and contraindications to MRI for those selected for scanning.

Study procedure

After a preliminary eligibility screen on the phone (Figure 1), patients will attend a baseline appointment with a clinically trained investigator (e.g, physiotherapist) at the local University Department. During the baseline appointment, the diagnosis of sciatica will be confirmed, and the prognostic variables will be assessed through a detailed set of clinical phenotyping as described below. Some patients will also undergo an MRI scan of their lumbar spine. We will then follow up patients over 1 year with monthly pain diaries (Appendix 1) and outcome will be measured at 3 (short-term) and 12 months (long-term). Published sciatica trajectories suggest that most improvement occurs within the first 3-4 months with little change up to 36 months.[20] Our time points should therefore give a comprehensive idea about short and long-term outcome, and are similar to other longitudinal sciatica cohorts thus facilitating cross-comparison.[8]

Outcome measures to define pain persistence

The final selection of our outcome measures has been guided by our patient advisory group and feedback from participants in the feasibility study. Pain persistence will be defined with the Sciatica Bothersomeness Index[21] and a numerical pain rating scale (0 no pain to 10 worst pain imaginable, primary outcomes). The Sciatica Bothersomeness Index (SBI) includes elements of leg pain as well as sensory and motor disturbances, thus providing a comprehensive measure of different sciatica symptoms. This index has shown good discrimination between self-reported successful and non-successful outcome in patients with sciatica[22] and has been favoured by our patient advisory group. In our feasibility study both outcome measures identified 38% of participants who developed persistent pain, which is in line with previous reports.[9] In line with recommendations, we will use continuous outcomes for statistical analyses. We may use dichotomisation to help data presentation in figures/tables. In this case, we will use a cut-off of >6.5 on the SBI , which has good validity to identify patients with unsuccessful sciatica outcome.[22]

We may also run analyses using secondary outcomes (e.g., disability using Oswestry Disability Index (ODI 2.1a)[23], self-perceived change using global rating of change scale (GROC)[24]).

Primary mechanism-based prognostic variables

1) Self-reported sensory profiling

See Table 1 for questionnaires. The Neuropathic Pain Symptom Inventory (NPSI) and PainDETECT will be used to determine sensory symptom clusters as previously reported.[25] Patients will be instructed to report the localisation of pain, paraesthesia and hypoesthesia on separate body charts by means of

For peer review only

pen-on-paper pain drawings (A4 sheets including ventral and dorsal view of female or male body). All drawings will be digitised and analysed using online software (https://syp.spslab.ch). The derived variables (i.e. extent and location) will be used to describe the symptoms associated with sciatica at the baseline. These have been shown to provide clues about central sensitisation[26 27]and may predict clinical outcome in other conditions.[28 29]

2) Somatosensory profiling

There is preliminary evidence that some quantitative sensory testing (QST) parameters may be prognostic in patients with a range of pain conditions including neuropathic pain.[15 16] The standardised and validated QST battery developed by the German Network for Neuropathic Pain (DFNS) will be used to reliably determine sensory function in different nerve fibres. Cold and warm detection thresholds (CDT, WDT; average of three repetitions) as well as cold and heat pain thresholds (CPT, HPT, average of three repetitions) and thermal sensory limen (TSL) including paradoxical heat sensations during three series of alternating cold and warm stimuli will be examined with a Thermotester (Somedic, Sweden, 25x50mm thermode). Mechanical detection thresholds (MDT) will be measured with von Frey hairs and mechanical pain thresholds (MPT) with weighted pin-prick stimulators (geometric mean of five series of ascending and descending stimuli). Mechanical pain sensitivity (MPS) will be examined with a numerical pain rating scale (0-100) during a shortened protocol of two sets of seven pseudo-random pin-prick stimulations.[30] To determine the presence of allodynia, two sets of three light touch stimulations with a cotton wisp, a cotton wool tip, and a standardized brush (Sense-lab) will be intermingled with these pin-prick stimulations. Pressure pain thresholds (PPT) will be evaluated with a manual algometer (Wagner Instruments, USA) and vibration detection threshold (VDT) with a Rydel Seiffer tuning fork (average of three repetitions). The wind-up ratio (WUR) will be determined as the mean numerical pain rating of three trains of 10 pin-prick stimuli divided by the mean rating of three single stimuli.

A shortened QST battery will first be conducted on the hand ipsilateral to the (most) symptomatic leg (CPT, HPT and MPT on dorsum of hand; PPT over thenar eminence) to determine the presence of widespread hyperalgesia. The full QST protocol will then be performed in the area of maximal pain in the affected leg where pervious work has shown QST changes in patients with 'sciatica'.[31]

We will use healthy control data to calculate Z-scores, where each individual parameter is related to its region-, age- and gender specific reference range. We will collect our own normative data, assisted by the provision of an existing QST dataset.[32] Using a previously published algorithm[13], patients will also be assigned one of the following somatosensory profiles 1) sensory loss 2) thermal hyperalgesia 3) mechanical hyperalgesia.

For peer review only

Further, we will include a conditioned pain modulation (CPM) paradigm to examine the efficacy of the descending pain modulatory system. Such dynamic QST protocols have shown most promising prognostic ability in other pain conditions.[15 16] Based on current recommendations[33], we will evaluate a sequential CPM paradigm using PPT over the thenar eminence of the dominant hand (test stimulus, average of 3 repetitions) and cold-water immersion of the non-dominant hand to the level of the wrist (conditioning stimulus). This combination has provided the most reliable and large magnitude CPM effects.[34] The water bath will be standardized to 4°C ± 2°C by adding ice. Patients are asked to report the intensity of pain experienced by cold water immersion from 0 (no pain) to 100 (worst pain imaginable). Once the pain reaches the cut-off of >40/100, or after a maximum of two minutes if this cut-off is not reached,[33 35] the participants will be asked to remove the hand from the water bath. The test stimulus will be repeated immediately thereafter. Cold water immersion is the most used CPM conditioning stimulus, is easy to implement and seems to be the most effective CPM paradigm.[36 37] PPT measurements are convenient, quickly measured and frequently used as a test stimulus.[38] A good to excellent intra-session reliability for CPM assessment with PPTs has been reported.[37 39]

3) Psychosocial profiles

There is a large body of evidence supporting the role of psychosocial factors in the persistence of pain and disability.[40 41] Therefore, we will assess psychosocial factors to examine their prognostic value in sciatica. The selection of specific measures of psychosocial factors drew upon existing evidence for their predictive utility in the context of other pain conditions, their theoretical relevance, and their psychometric properties including content validity.[42] We will have a two-level approach to assessment that includes general or "transdiagnostic" psychosocial factors and condition/sciatica-specific factors (Table 1). The transdiagnostic factors include symptoms of depression and general anxiety, sleep disturbance, and fatigue (all measured with their respective PROMIS SF8a tools[43]), trauma history, pain-related worry ("Pain Catastrophizing Scale")[44] and personality (Ten Item Personality Inventory[45]). In addition to transdiagnostic psychosocial risk factors, we have included several measures of potential protective factors (ie, optimism, State Optimism Measure[46]; social support, PROMIS SF4a instrumental and emotional Support; and social role participation, PROMIS SF8a) to provide a more holistic assessment. To assess cognitions specific to the context of sciatica, we developed a novel item set that was primarily adapted from the revised Illness Perception Questionnaire (Appendix 2).[47] Patient partners provided extensive feedback to develop and refine the sciatica-specific adaptation of these items. We have also included a measure of stigma[48] in relation to sciatica.

4) Blood inflammatory markers

We will sample blood by cubital venepuncture into BD Vacutainer SST and serum clot activator tubes (gold and red cap, BD, Wokingham United Kingdom). The time of last meal will be recorded. Thirty

For peer review only

minutes after venepuncture, the blood will be centrifuged at 1.3g for 10 minutes at 4°C (gold cap for protein analysis) and at room temperature (red cap tubes for metabolomics). The serum fraction will be immediately aliquoted and stored at -80°C for batch processing.

We will use complimentary protein/metabolomics analysis to evaluate serum inflammatory markers related to inflammation and neuropathic pain. Protein analysis will utilise a custom-made electro-chemiluminescent multiplex biomarkers assays (MSD) available at Oxford. These plates contain 17 cytokines/chemokines including candidates of interest derived in our previous work (e.g., IL-4, IL-9, IL-6).[49] Patient samples will be run in duplicate and normalised to standard curves.

Metabolomic analyses will be carried out using a state-of-the-art, high-field 700 MHz NMR spectrometer equipped with TCI cryoprobe (Department of Chemistry, University of Oxford), as previously described.[50] Quality control samples will be randomly spread throughout the run for standardisation and internal reference standards will allow absolute concentrations of inflammatory markers (N-acetylated glycoprotein species, serum lipoproteins,) along with energy and TCA-cycle metabolites to be determined.

Additional phenotypic data

Demographics and medical information

We will also collect basic demographic data (e.g., age, gender, ethnicity, profession, working status, perception of household income, years of school attendance) and medical information (e.g., most affected side, previous history of back pain or sciatica, number of previous episodes, duration of current episode, family history of pain, current and past medical history including current and past medications and their effectiveness, trialled treatments, results of previous imaging, smoking and alcohol intake, Appendix 3).

Clinical Examination

We will also perform a clinical examination (Appendix 4). We will document height, weight and hip/waist circumference. We will record findings from a bedside neurological screening examination of the lower limbs. This includes myotomal testing from lumbar levels L2-S1, patellar and achilles tendon reflexes, as well as mapping of sensory loss to light touch and pin prick on body charts. We will check for upper motor neurone signs (exclusion criteria) using Hoffmann's test, Babinski, inverted supinator sign and observation of tandem walk.[51] Patients will go through a warning sign checklist for suspected cauda equina syndrome (exclusion criteria).[52]

We will perform the straight leg raise and slump test as well as femoral slump if indicated (e.g., presentation suggesting upper lumbar involvement).[53] These tests for nerve mechanosensitivity will

For peer review only

be deemed positive if they 1) reproduce at least partially the patients' symptoms and 2) if structural differentiation through either foot dorsiflexion or cervical flexion changes the symptoms. [54] We will further record the presence of lumbar shifts, active range of motion restrictions in lower back and hip including whether these movements provoke back or leg symptoms. Pain provocation upon posterior anterior intervertebral movement palpation of the lumbar segments L1-L5 will be recorded (Grade IV unless pain provocation occurs earlier).

At the end of the baseline appointment, the assessor will rate the certainty of neuropathic leg pain as unlikely, possible, probable or definite according to the updated neuropathic pain grading system.[55] They will also assign patients to one or several of the following subgroups described elsewhere[56]: radiculopathy (true neurological deficit), radicular pain, neural mechanosensitivity or somatic referred pain.

Self-reported questionnaires

We will also collect the following additional questionnaires to describe our patient population: ODI[57] (separate questionnaires for back and leg), Keele Start Back tool, [58] EQ-5D,[59] and a monthly pain diary (Appendix 3).

Magnetic resonance neurography (MRN)

We will perform MRN in a subset of n=100 patients with sciatica and n=44 healthy matched controls to identify moderate effects[23] (d=0.52, alpha=0.05, 80% power). Eligible patients (e.g., MRI safety) will be consecutively recruited for scanning until numbers are reached.

We will perform advanced MRN optimised to visualise lumbar nerve root macro- and microstructure at 3 Tesla using a dedicated 18-channel phased array spine coil (Siemens, UK). The protocol includes multi-shell (b=700 and 1500 s/mm2) DTI scans, high resolution anatomic scans with optimised T1 and T2 weighted contrasts, and a T2 mapping scan (Appendix 5). The data analysis will be performed using FSL tools including TOPUP[60 61] and EDDY [62-64] for the correction of images' distortions and subject movements, DTIFIT[65] for the fitting of diffusion tensor model, and FLIRT[66 67] for the registration of diffusion metrics and anatomic images. Measures including fractional anisotropy, mean/axial/radial diffusivity and T2 maps will be obtained within regions of interest in lumbar nerve roots (affected and unaffected sides) and averaged over multiple slices as we have optimised before.[68]

Cohort harmonisation

The FORECAST cohort is harmonised with the Advanced Pain Discovery Platform funded PAINSTORM consortium, and therefore includes additional measures that will allow data integration (e.g., blood collection for genetic analyses, skin biopsies in the maximal pain area, DN4,[69] Michigan Neuropathy Screening Instrument,[70] Chronic pain grade,[71] Brief pain inventory[72] (pain intensity

For peer review only

items), a section where patients can tell us more about their pain and circumstances in their own words including how they would describe their pain to their friends/family or work colleagues, as well as their feelings about their financial situation and its impact on their situation. This harmonisation may also enable external validation of the FORECAST findings in other neuropathies.

Data analysis plan

Statistical methods will follow STROBE guidelines[17] and the TRIPOD statement for transparent reporting of a multivariable prediction model for individual prognosis or diagnosis.[18]

Participants' baseline characteristics (e.g., demographics, disability using (ODI), medical co-morbidities) and their clinical course (primary and secondary outcomes, ODI) will be described for short (3 months) and long-term time-points (12 months).

To identify and characterise mechanism-based subgroups in patients with acute/subacute sciatica and use distance-based clustering algorithms efficiently we first need to address the high dimensionality – modest sample size of the dataset. Thus, we will first carry out a Principal Component Analysis (PCA) to summarise and reduce the dimensionality of the dataset while preserving as much variability as possible. Then we will use algorithmic centroid (k-means) and hierarchical clustering based on the Euclidean distance between principal dimensions to identify sub-groups of patients sharing high phenotypic similarities. The optimal number of clusters will be determined using the gap statistic and the elbow of the within/between clusters variance plot. Consequently, we will perform hypothesis testing to assess group differences on the original variables between participants assigned to different clusters. All omnibus tests will be followed-up by the appropriate post-hoc test.

To investigate factors that predict pain persistence in people with sciatica we will use variable selection techniques followed by predictive modelling. First, we will perform filtering of the original variables by calculating the univariate associations (coefficients, 95% CI, p-values) between variables and the outcome and between each other. We will select a subset of uncorrelated variables that are associated with the outcome and use them as input features in machine learning algorithms for high dimensional, small datasets that will allow us to identify the most powerful predictors and assess model selection/predictive accuracy. During pre-processing, missing data will be examined, the mechanism of missingness will be inferred using hypothesis testing and visually assessed using a matrix of boxplots for all pairs of variables and the outcome, and if appropriate multiple imputation by chained equations will be used. Drawing from machine learning techniques for high dimensional small datasets we will use re-sampling and validation in the form of repeated cross-validation to perform a complete variable profiling to identify the most powerful predictors. Multivariate Adaptive Regression Splines (MARS) with built-in feature selection and Decision Tree models known to work well on low sample sizes will be trained to predict the 3-month and 1-year outcome. Model performance will be estimated using 5-

For peer review only

times repeated 10-fold cross-validation and compared to models trained on surrogate data.[73] The latter benchmarking technique is appropriate for small datasets, where holding out a subset of data before the analysis to be used as a pseudo-independent test set is impossible. Instead, an artificial – surrogate dataset, preserving the descriptive statistics but not any of the potentially real associations between the variables and the outcome of the original dataset, will be created and the performance of models trained on the actual and surrogate dataset will be compared. Models' predictive performance will be reported alongside variable importance rankings. Model selection will be done to maximise the Mathews Correlation Coefficient for dichotomised outcomes and to minimise the Root Mean Square Error (RMSE) for continuous outcomes during cross-validation. Scalar metric estimations of predictive performance including accuracy (binomial test p-value against the majority class prevalence), balanced accuracy and the area under the precision/recall curve will be reported alongside their 95% CI. Predictor importance will be assessed using model specific techniques, i.e., the reduction in performance estimated by cross-validation when each predictor is removed for MARS and node impurity for tree-based methods. Variables' influence on the predicted outcome both at the global and individual level will be quantified by the Partial Dependence Plots and Individual Conditional Expectation[74] respectively. These will show the average marginal effect on the prediction given a certain value of a predictor variable and provide model interpretability.

Sample size estimation

QST sensory profiles: Published sample size guidelines for QST clustering in peripheral nerve injury[75] suggest that for strong effects (effect size = 0.7) a sample size of <180 patients will produce a subpopulation with thermal and mechanical hyperalgesia large enough to conduct a study with 80% power, at an alpha 0.05. To calculate QST z-scores, at least 8 controls are required for each area and age decade.[76] Our feasibility study included patients of 7 age decades with 3 main pain areas. We will therefore need n=168 controls.

k-means and hierarchical clustering after PCA: Using the 2 first principal dimensions for 3 variable domains (self-reported profiling, QST, inflammatory markers) we will need 2^6=64 patients to perform k-means clustering with adequate power.[77]

Algorithmic cluster analysis: Assuming k=4 clusters, we will be able to identify moderate effects (effect size = 0.25) with an one-way ANOVA between 4 groups at an alpha level of 0.05, 80% power.

Predictor profiling: FORECAST aims to identify prognostic factors (the first step in the PROGRESS framework[78]) rather than developing a clinical prognostic tool or individual risk model which requires much larger sample sizes. We will use robust algorithms that include feature selection, and we will assess model performance using methods developed for small datasets and robust metrics. As this part is an exploratory analysis that could shape future hypotheses and validation studies, our sample size is

For peer review only

adequate. Given the anticipated sample size ratios with chronic (180*30%=54) and resolved sciatica (180*70%=126) and accounting for 15% attrition (see feasibility study), we will be able to identify moderate effects (effect size = 0.5) using a two-tailed Wilcoxon-Mann-Whitney test (power 81%, alpha 0.05).

Ethics and dissemination

The FORECAST study has received ethical approval (South Central Oxford C, 18/SC/0263). All participants will provide informed written consent before participating in the study.

The dissemination strategy will be strongly guided by our PPIE activities (see below). This will be based on co-productions between patient partners and academics and will involve publication of findings in scientific journals, presentations at conferences, media pieces (mainstream and social media) as well as communication through charity partners.

Data will be made publicly available on the ALLEVIATE data hub (https://alleviate.ac.uk) and remaining bio-samples will be on-boarded to the Imperial Biobank. The data and samples will continue to be linked and will be available for future studies.

Patient and Public Involvement and Engagement (PPIE) and Dissemination of Findings

The FORECAST team consists of equal partners including patient partners, clinicians and researchers. Our aims have been shaped by the needs of people living with sciatica to ensure we address unmet needs. The PPIE plans will be shaped by the following members of FORECAST: 1) Inclusion of two patient partners as co-investigators (CR, CP). They will contribute as equal partners on the investigator team. 2) PPIE lead with extensive experience in involving patients' voices in research (KRM). 3) diverse patient advisory group (PAG) consisting of six individuals with a lived experience of sciatica. Our patient partners and PAG provided early input to the original grant application and identification of key research activities within the project, particularly around including the feasibility work (e.g., acceptability of testing and study procedures), study design (e.g., selection of primary outcome measure) and strongly informed the writing of our funding application (e.g., lay summary). We will continue to work closely with people with lived experience of sciatica as we undertake this study, and our PPIE strategy will continue to be implemented throughout the lifetime of FORECAST. We will seek the perspective and guidance of our patient partners and PAG members on matters including, but not limited to participant recruitment and retention; barriers/facilitators of participation among seldom heard populations; data analysis and sensemaking of findings, organisation and co-

For peer review only

production of workshops, dissemination materials, and public engagement activities. This will ensure that the patient perspective has been considered at all stages throughout the project.

We will also work closely with our patient partners and advisors on engagement and dissemination activities. This may include, but is not limited to, co-producing newsletters, lay summaries, website content, infographics, animated videos, and podcasts, as well as engagement activities to bring the project into a public sphere. We plan to work closely with the PAINSTORM research team and patient partners, as well as other national and international pain and sciatica groups to promote the study and its subsequent findings. This would allow us to reflect on the way the conclusions are presented and identify any gaps which might lead to further research in the topic area. We also plan to hold conversations with our patient partners and PAG regarding planning and undertaking academic dissemination activities (e.g., engagement with policy stakeholders, conference abstracts/presentations, manuscript preparation/publication). All individuals who contribute to this PPI advisory group will receive payment in accordance with current INVOLVE guidelines.

For peer review only

Acknowledgement

The authors wish to thank all participants of the feasibility study who have helped shape the protocol of the FORECAST study. The input of Prof Irene Tracey in the design of the study is also gratefully acknowledged.

Author contributions:

ABS has conceived the FORECAST study and acquired funding with the support of all FORECAST collaborators. LR is coordinating the FORECAST study and SK is supporting data collection. LH coordinated the feasibility study. FP assisted in the design of the blood marker analysis and will perform all metabolomics data acquisition, processing, and analyses. WS and GC led on the psychosocial profiling. MT, SC, DN, and SA contributed to the development of the MRI sequences and/or will perform MRI-related analyses. CP, CR and KM are responsible for the PPIE. BT has provided input into the QST assessment and will provide normative QST datasets. MB will provide the SYP body diagram software and run the pain drawing analyses. JF will provide clinical oversight. GB performed sample size analyses and will be responsible for overall data integration and analysis. All authors have contributed to the study design and write up of the protocol and approved the final version.

Funding statement:

This project is funded by UKRI and Versus Arthritis as part of the UKRI Strategic Priorities Fund (SPF) Advanced Pain Discovery Platform (APDP), a co-funded initiative by UKRI (MRC, BBSRC, ESRC), Versus Arthritis, the Medical Research Foundation and Eli Lilly and Company Ltd (Grant MR/W027003/1).

ABS is supported by a Wellcome Trust Clinical Career Development Fellowship (222101/Z/20/Z).

BT received funding to collect normative QST data from the Government of Western Australia, Department of Health and the Raine Medical Research Foundation, the Sir Charles Gairdner Hospital and Osborne Park Health Care Group Research Advisory Committee Grant (RAC 2016-17/015) and the Charlies Foundation for Research, Arthritis Australia (The Eventide Homes Grant) and School of Physiotherapy and Exercise Science, Curtin University.

WS is partly funded through the National Institute for Health and Care Research (NIHR) Biomedical Research Centre at the South London and Maudsley NHS Foundation Trust and King's College London.

FP is funded by a Dorothy Hodgkin Career Development Fellowship in Chemistry in association with Somerville College.

GB is supported by the Wellcome Trust, grant reference 223149/Z/21/Z and Diabetes UK, grant reference 19/0005984.

For peer review only

GC and KRM are partly funded by UKRI and Versus Arthritis as part of the Advanced Pain Discovery Platform (APDP) PAINSTORM (MR/W002388/1).
LH was supported by a preparatory research fellowship from the NIHR Biomedical Research Centre Oxford, based at Oxford University Hospital NHS Foundation Trust, Oxford. The research is supported by the National Institute for Health Research (NIHR) Oxford Health Biomedical Research Centre (BRC). The views expressed are those of the authors and not necessarily those of the NHS, the NIHR or the Department of Health and Social Care

Data statement: Data will be made publicly available on the ALLEVIATE data hub (https://alleviate.ac.uk) and remaining bio-samples will be on-boarded to the Imperial Biobank. The data and samples will continue to be linked and will be available for future studies.

Competing interest statement: The authors declare no competing interests.

For peer review only

Table 1: Questionnaires

**Questionnaires** *primary outcome **secondary outcome		FORECAST patients		Healthy volunteers	PAINSTORM DATASET
		BASELINE	FOLLOW UP	BASELINE	EXTENDED
FORECAST outcomes	Sciatica Bothersomeness Index (SBI)[21]	X	X*		
	Numerical Pain Rating Scale - previous 2 weeks (worst, least, average for leg /back pain)	X	X*		
	Global Rating of Change Scale		X**		
Neuropathy/Neuropathic Pain	PainDETECT[79]	X	X		X
	Neuropathic Pain Symptom Inventory (NPSI)[80]	X	X	X	X
	DN4[69]		X		X
	Michigan Neuropathy Screening Instrument (MNSI)[70]				X
Pain location, severity	Pain location - list of sites, body chart	X	X	X	X
	Monthly pain diary	X	X		
	Chronic Pain Grade (CPG)[71]				X
	Brief Pain Inventory (BPI)[72]				X
Disability	Oswestry Disability Index (ODI 2.1a)[57] – Leg Pain	X	X**		
	Oswestry Disability Index (ODI 2.1a)[57] – Low Back Pain	X	X		
	Oswestry Disability Index (ODI 2.1a)[57] - combined leg and back pain			X	
Risk	Keele STarT Back tool[58]	X			
Lifestyle	International Physical Activity Questionnaire (IPAQ, long version)[81]	X	X	X	X

For peer review only

		FORECAST patients		Healthy volunteers	PAINSTORM DATASET
		BASELINE	FOLLOW UP	BASELINE	EXTENDED
Quality of life	EQ-5D-5L (v1.2)[59]	X	X	X	X
Psychosocial Questionnaires	PROMIS SF8a – Ability to participate in social roles and activities[43] (v1.0)	X	X	X	X
	Pain Catastrophising Scale (PCS)[44]	X	X	X	X
	PROMIS SF8-a – Depression and Anxiety[43] (v1.0)	X	X	X	X
	Adverse Childhood Events (ACEs) (none, 1, 2, >2)	X		X	X
	Prolonged hospitalisation for life threatening condition (yes/no)	X		X	X
	PROMIS SF8a – Sleep Disturbance[43] (v1.0)	X	X	X	X
	PROMIS SF8a – Fatigue[43] (v1.0)	X	X	X	X
	PROMIS SF4a-instrumental support[43] (v1.0)	X	X	X	X
	PROMIS SF4a – Emotional Support[43] (v1.0)	X	X	X	X
	Ten Item Personality Index (TIPI)[45]	X		X	X
	State Optimism Measure (SOM-7)	X	X	X	X
	Illness Perception Questionnaire (IPQ-R)[82]	X			
	Sciatica Perception Questionnaire (SPQ)	X	X		
	Stigma Scale for Chronic Illnesses (SSCI) - modified[48]	X	X		
	"in your own words" (impact on social and financial situation)				X

For peer review only

References

1. Wu A, March L, Zheng X, et al. Global low back pain prevalence and years lived with disability from 1990 to 2017: estimates from the Global Burden of Disease Study 2017. *Ann Transl Med* 2020;8(6):299. doi: 10.21037/atm.2020.02.175 [published Online First: 2020/05/02]
2. Kleinman N, Patel AA, Benson C, et al. Economic burden of back and neck pain: effect of a neuropathic component. *Population health management* 2014;17(4):224-32. doi: 10.1089/pop.2013.0071
3. Centre NG. NICE guideline NG59: Low back pain and sciatica in over 16s: assessment and management. In: UK NIfHaCE, ed., 2016.
4. Pinto RZ, Maher CG, Ferreira ML, et al. Drugs for relief of pain in patients with sciatica: systematic review and meta-analysis. *BMJ* 2012;344:e497. doi: 10.1136/bmj.e497 [published Online First: 2012/02/15]
5. Dove L, Jones G, Kelsey LA, et al. How effective are physiotherapy interventions in treating people with sciatica? A systematic review and meta-analysis. *Eur Spine J* 2022 doi: 10.1007/s00586-022-07356-y [published Online First: 20221229]
6. Haugen AJ, Brox JI, Grovle L, et al. Prognostic factors for non-success in patients with sciatica and disc herniation. *BMC Musculoskelet Disord* 2012;13:183. doi: 10.1186/1471-2474-13-183
7. Iversen T, Solberg TK, Wilsgaard T, et al. Outcome prediction in chronic unilateral lumbar radiculopathy: prospective cohort study. *BMC Musculoskelet Disord* 2015;16:17. doi: 10.1186/s12891-015-0474-9 [published Online First: 2015/04/19]
8. Konstantinou K, Dunn KM, Ogollah R, et al. Prognosis of sciatica and back-related leg pain in primary care: the ATLAS cohort. *Spine J* 2018;18(6):1030-40. doi: 10.1016/j.spinee.2017.10.071 [published Online First: 2017/11/28]
9. Vroomen PC, de Krom MC, Slofstra PD, et al. Conservative treatment of sciatica: a systematic review. *J Spinal Disord* 2000;13(6):463-9. [published Online First: 2001/01/02]
10. Weber H, Holme I, Amlie E. The natural course of acute sciatica with nerve root symptoms in a double-blind placebo-controlled trial evaluating the effect of piroxicam. *Spine (Phila Pa 1976)* 1993;18(11):1433-8. [published Online First: 1993/09/01]
11. Ashworth J, Konstantinou K, Dunn KM. Prognostic factors in non-surgically treated sciatica: a systematic review. *BMC Musculoskelet Disord* 2011;12:208. doi: 10.1186/1471-2474-12-208 [published Online First: 2011/09/29]
12. Verwoerd AJ, Luijsterburg PA, Lin CW, et al. Systematic review of prognostic factors predicting outcome in non-surgically treated patients with sciatica. *Eur J Pain* 2013;17(8):1126-37. doi: 10.1002/j.1532-2149.2013.00301.x
13. Baron R, Maier C, Attal N, et al. Peripheral neuropathic pain: a mechanism-related organizing principle based on sensory profiles. *Pain* 2017;158(2):261-72. doi: 10.1097/j.pain.0000000000000753
14. Baron R, Wasner G, Binder A. Chronic pain: genes, plasticity, and phenotypes. *Lancet Neurol* 2012;11(1):19-21. doi: 10.1016/S1474-4422(11)70281-2 [published Online First: 2011/12/17]
15. Petersen KK, Vaegter HB, Stubhaug A, et al. The predictive value of quantitative sensory testing: a systematic review on chronic postoperative pain and the analgesic effect of pharmacological therapies in patients with chronic pain. *Pain* 2021;162(1):31-44. doi: 10.1097/j.pain.0000000000002019 [published Online First: 2020/07/24]
16. Georgopoulos V, Akin-Akinyosoye K, Zhang W, et al. Quantitative sensory testing and predicting outcomes for musculoskeletal pain, disability, and negative affect: a systematic review and meta-analysis. *Pain* 2019;160(9):1920-32. doi: 10.1097/j.pain.0000000000001590 [published Online First: 2019/05/03]
17. von Elm E, Altman DG, Egger M, et al. The Strengthening the Reporting of Observational Studies in Epidemiology (STROBE) statement: guidelines for reporting observational studies. *Lancet* 2007;370(9596):1453-7. doi: 10.1016/S0140-6736(07)61602-X [published Online First: 2007/12/08]

For peer review only

18. Collins GS, Reitsma JB, Altman DG, et al. Transparent reporting of a multivariable prediction model for individual prognosis or diagnosis (TRIPOD): the TRIPOD statement. *BMJ* 2015;350:g7594. doi: 10.1136/bmj.g7594 [published Online First: 2015/01/09]
19. Stynes S, Konstantinou K, Ogollah R, et al. Clinical diagnostic model for sciatica developed in primary care patients with low back-related leg pain. *PLoS ONE* 2018;13(4):e0191852. doi: 10.1371/journal.pone.0191852 [published Online First: 2018/04/06]
20. Harrisson SA, Ogollah R, Dunn KM, et al. Prevalence, Characteristics, and Clinical Course of Neuropathic Pain in Primary Care Patients Consulting With Low Back-related Leg Pain. *Clin J Pain* 2020;36(11):813-24. doi: 10.1097/AJP.0000000000000879 [published Online First: 2020/08/26]
21. Patrick DL, Deyo RA, Atlas SJ, et al. Assessing health-related quality of life in patients with sciatica. *Spine (Phila Pa 1976)* 1995;20(17):1899-908; discussion 909. [published Online First: 1995/09/01]
22. Haugen AJ, Grovle L, Brox JI, et al. Estimates of success in patients with sciatica due to lumbar disc herniation depend upon outcome measure. *Eur Spine J* 2011;20(10):1669-75. doi: 10.1007/s00586-011-1809-3 [published Online First: 2011/04/26]
23. Fairbank J. Oswestry Disability Index: ePROVIDE; 2022 [Available from: https://eprovide.mapi-trust.org/instruments/oswestry-disability-index2022.
24. Jaeschke R, Singer J, Guyatt GH. Measurement of health status. Ascertaining the minimal clinically important difference. *Control Clin Trials* 1989;10(4):407-15. [published Online First: 1989/12/01]
25. Mahn F, Hullemann P, Gockel U, et al. Sensory symptom profiles and co-morbidities in painful radiculopathy. *PLoS ONE* 2011;6(5):e18018. doi: 10.1371/journal.pone.0018018
26. Willett MJ, Siebertz M, Petzke F, et al. The Extent of Pain Is Associated With Signs of Central Sensitization in Patients With Hip Osteoarthritis. *Pain Pract* 2020;20(3):277-88. doi: 10.1111/papr.12851 [published Online First: 2019/10/31]
27. Lluch Girbes E, Duenas L, Barbero M, et al. Expanded Distribution of Pain as a Sign of Central Sensitization in Individuals With Symptomatic Knee Osteoarthritis. *Phys Ther* 2016;96(8):1196-207. doi: 10.2522/ptj.20150492 [published Online First: 2016/03/05]
28. Evans DW, Rushton A, Middlebrook N, et al. Estimating Risk of Chronic Pain and Disability Following Musculoskeletal Trauma in the United Kingdom. *JAMA Netw Open* 2022;5(8):e2228870. doi: 10.1001/jamanetworkopen.2022.28870 [published Online First: 2022/08/27]
29. Alter BJ, Anderson NP, Gillman AG, et al. Hierarchical clustering by patient-reported pain distribution alone identifies distinct chronic pain subgroups differing by pain intensity, quality, and clinical outcomes. *PLoS ONE* 2021;16(8):e0254862. doi: 10.1371/journal.pone.0254862 [published Online First: 2021/08/05]
30. Pascal MMV, Themistocleous AC, Baron R, et al. DOLORisk: study protocol for a multi-centre observational study to understand the risk factors and determinants of neuropathic pain. *Wellcome Open Res* 2018;3:63. doi: 10.12688/wellcomeopenres.14576.2 [published Online First: 2019/02/16]
31. Tampin B, Slater H, Jacques A, et al. Association of quantitative sensory testing parameters with clinical outcome in patients with lumbar radiculopathy undergoing microdiscectomy. *Eur J Pain* 2020;24(7):1377-92. doi: 10.1002/ejp.1586 [published Online First: 2020/05/10]
32. Zhu GC, Bottger K, Slater H, et al. Concurrent validity of a low-cost and time-efficient clinical sensory test battery to evaluate somatosensory dysfunction. *Eur J Pain* 2019;23(10):1826-38. doi: 10.1002/ejp.1456 [published Online First: 2019/07/22]
33. Yarnitsky D, Bouhassira D, Drewes AM, et al. Recommendations on practice of conditioned pain modulation (CPM) testing. *Eur J Pain* 2015;19(6):805-6. doi: 10.1002/ejp.605 [published Online First: 2014/10/21]
34. Imai Y, Petersen KK, Morch CD, et al. Comparing test-retest reliability and magnitude of conditioned pain modulation using different combinations of test and conditioning stimuli. *Somatosens Mot Res* 2016;33(3-4):169-77. doi: 10.1080/08990220.2016.1229178 [published Online First: 2016/09/22]

For peer review only

35. Nir RR, Granovsky Y, Yarnitsky D, et al. A psychophysical study of endogenous analgesia: the role of the conditioning pain in the induction and magnitude of conditioned pain modulation. *Eur J Pain* 2011;15(5):491-7. doi: 10.1016/j.ejpain.2010.10.001 [published Online First: 2010/11/03]
36. Aparecida da Silva V, Galhardoni R, Teixeira MJ, et al. Not just a matter of pain intensity: Effects of three different conditioning stimuli on conditioned pain modulation effects. *Neurophysiol Clin* 2018;48(5):287-93. doi: 10.1016/j.neucli.2018.06.078 [published Online First: 2018/06/30]
37. Kennedy DL, Kemp HI, Ridout D, et al. Reliability of conditioned pain modulation: a systematic review. *Pain* 2016;157(11):2410-19. doi: 10.1097/j.pain.0000000000000689 [published Online First: 2016/10/19]
38. Pud D, Granovsky Y, Yarnitsky D. The methodology of experimentally induced diffuse noxious inhibitory control (DNIC)-like effect in humans. *Pain* 2009;144(1-2):16-9. doi: 10.1016/j.pain.2009.02.015 [published Online First: 2009/04/11]
39. Lewis GN, Rice DA, McNair PJ. Conditioned pain modulation in populations with chronic pain: a systematic review and meta-analysis. *J Pain* 2012;13(10):936-44. doi: 10.1016/j.jpain.2012.07.005 [published Online First: 2012/09/18]
40. Giusti EM, Lacerenza M, Manzoni GM, et al. Psychological and psychosocial predictors of chronic postsurgical pain: a systematic review and meta-analysis. *Pain* 2021;162(1):10-30. doi: 10.1097/j.pain.0000000000001999 [published Online First: 2020/07/23]
41. Hruschak V, Cochran G. Psychosocial predictors in the transition from acute to chronic pain: a systematic review. *Psychol Health Med* 2018;23(10):1151-67. doi: 10.1080/13548506.2018.1446097 [published Online First: 2018/03/02]
42. Crombez G, De Paepe AL, Veirman E, et al. Let's talk about pain catastrophizing measures: an item content analysis. *Peerj* 2020;8 doi: ARTN e8643
10.7717/peerj.8643
43. Cella D, Yount S, Rothrock N, et al. The Patient-Reported Outcomes Measurement Information System (PROMIS): progress of an NIH Roadmap cooperative group during its first two years. *Med Care* 2007;45(5 Suppl 1):S3-S11. doi: 10.1097/01.mlr.0000258615.42478.55
44. Sullivan MJL, Bishop SR, Pivik J. The Pain Catastrophizing Scale: Development and Validation. *Psychol Assess* 1995;7:4.
45. Gosling SD, Rentfrow PJ, Swann WB. A very brief measure of the Big-Five personality domains. *Journal of Research in Personality* 2003;37(6):504-28. doi: 10.1016/S0092-6566(03)00046-1
46. Millstein RA, Chung WJ, Hoeppner BB, et al. Development of the State Optimism Measure. *Gen Hosp Psychiatry* 2019;58:83-93. doi: 10.1016/j.genhosppsych.2019.04.002 [published Online First: 2019/04/27]
47. Moss-Morris R, Weinman J, Petrie KJ, et al. The revised Illness Perception Questionnaire (IPQ-R). *Psychology & Health* 2002;17(1):1-16. doi: 10.1080/08870440290001494
48. Molina Y, Choi SW, Cella D, et al. The Stigma Scale for Chronic Illnesses 8-Item Version (SSCI-8): Development, Validation and Use Across Neurological Conditions. *International Journal of Behavioral Medicine* 2013;20(3):450-60. doi: 10.1007/s12529-012-9243-4
49. Sandy-Hindmarch O, Bennett DL, Wiberg A, et al. Systemic inflammatory markers in neuropathic pain, nerve injury and recovery. *Pain* 2021 doi: 10.1097/j.pain.0000000000002386 [published Online First: 2021/07/06]
50. Probert F, Yeo T, Zhou Y, et al. Integrative biochemical, proteomics and metabolomics cerebrospinal fluid biomarkers predict clinical conversion to multiple sclerosis. *Brain Commun* 2021;3(2):fcab084. doi: 10.1093/braincomms/fcab084 [published Online First: 2021/05/18]
51. Cook C, Brown C, Isaacs R, et al. Clustered clinical findings for diagnosis of cervical spine myelopathy. *J Man Manip Ther* 2010;18(4):175-80. doi: 10.1179/106698110X12804993427045 [published Online First: 2011/12/02]
52. Greenhalgh S, Finucane L, Mercer C, et al. Assessment and management of cauda equina syndrome. *Musculoskelet Sci Pract* 2018;37:69-74. doi: 10.1016/j.msksp.2018.06.002 [published Online First: 2018/06/25]
53. Butler DS. The sensitive nervous system. Adelaide: NOIgroup publications 2000.

For peer review only

54. Nee RJ, Jull GA, Vicenzino B, et al. The validity of upper-limb neurodynamic tests for detecting peripheral neuropathic pain. *J Orthop Sports Phys Ther* 2012;42(5):413-24. doi: 10.2519/jospt.2012.3988 [published Online First: 2012/03/10]
55. Finnerup NB, Haroutounian S, Kamerman P, et al. Neuropathic pain: an updated grading system for research and clinical practice. *Pain* 2016;157(8):1599-606. doi: 10.1097/j.pain.0000000000000492
56. Schmid AB, Tampin B. Spinally Referred Back and Leg Pain. In: Spine ISftSotL, ed. Lumbar Spine Online Textbook. http://www.wheelessonline.com/ISSLS/section-10-chapter-10-spinally-referred-back-and-leg-pain/2018.
57. Fairbank JC, Pynsent PB. The Oswestry Disability Index. *Spine (Phila Pa 1976)* 2000;25(22):2940-52; discussion 52.
58. Hill JC, Dunn KM, Main CJ, et al. Subgrouping low back pain: a comparison of the STarT Back Tool with the Orebro Musculoskeletal Pain Screening Questionnaire. *Eur J Pain* 2010;14(1):83-9. doi: 10.1016/j.ejpain.2009.01.003 [published Online First: 2009/02/19]
59. Group TE. EuroQol-a new facility for the measurement of health-related quality of life. *Health Policy* 1990;16(3):199-208.
60. Andersson JL, Skare S, Ashburner J. How to correct susceptibility distortions in spin-echo echo-planar images: application to diffusion tensor imaging. *Neuroimage* 2003;20(2):870-88. doi: 10.1016/S1053-8119(03)00336-7
61. Smith SM, Jenkinson M, Woolrich MW, et al. Advances in functional and structural MR image analysis and implementation as FSL. *Neuroimage* 2004;23 Suppl 1:S208-19. doi: 10.1016/j.neuroimage.2004.07.051
62. Andersson JLR, Sotiropoulos SN. An integrated approach to correction for off-resonance effects and subject movement in diffusion MR imaging. *Neuroimage* 2016;125:1063-78. doi: 10.1016/j.neuroimage.2015.10.019 [published Online First: 20151020]
63. Andersson JLR, Graham MS, Drobnjak I, et al. Towards a comprehensive framework for movement and distortion correction of diffusion MR images: Within volume movement. *Neuroimage* 2017;152:450-66. doi: 10.1016/j.neuroimage.2017.02.085 [published Online First: 20170308]
64. Andersson JLR, Graham MS, Drobnjak I, et al. Susceptibility-induced distortion that varies due to motion: Correction in diffusion MR without acquiring additional data. *Neuroimage* 2018;171:277-95. doi: 10.1016/j.neuroimage.2017.12.040 [published Online First: 20171224]
65. Sotiropoulos SN, Hernandez-Fernandez M, Vu AT, et al. Fusion in diffusion MRI for improved fibre orientation estimation: An application to the 3T and 7T data of the Human Connectome Project. *Neuroimage* 2016;134:396-409. doi: 10.1016/j.neuroimage.2016.04.014 [published Online First: 20160409]
66. Jenkinson M, Smith S. A global optimisation method for robust affine registration of brain images. *Med Image Anal* 2001;5(2):143-56.
67. Jenkinson M, Bannister P, Brady M, et al. Improved optimization for the robust and accurate linear registration and motion correction of brain images. *Neuroimage* 2002;17(2):825-41.
68. Schmid AB, Campbell J, Hurley SA, et al. Feasibility of Diffusion Tensor and Morphologic Imaging of Peripheral Nerves at Ultra-High Field Strength. *Invest Radiol* 2018;53(12):705-13. doi: 10.1097/RLI.0000000000000492 [published Online First: 2018/07/07]
69. Bouhassira D, Attal N, Alchaar H, et al. Comparison of pain syndromes associated with nervous or somatic lesions and development of a new neuropathic pain diagnostic questionnaire (DN4). *Pain* 2005;114(1-2):29-36. doi: 10.1016/j.pain.2004.12.010 [published Online First: 2005/03/01]
70. Feldman EL, Stevens MJ, Thomas PK, et al. A practical two-step quantitative clinical and electrophysiological assessment for the diagnosis and staging of diabetic neuropathy. *Diabetes Care* 1994;17(11):1281-9.
71. Dixon D, Pollard B, Johnston M. What does the chronic pain grade questionnaire measure? *Pain* 2007;130(3):249-53. doi: 10.1016/j.pain.2006.12.004
72. Cleeland CS, Ryan KM. Pain assessment: global use of the Brief Pain Inventory. *Ann Acad Med Singap* 1994;23(2):129-38. [published Online First: 1994/03/01]

For peer review only

73. Hirata Y, Katori Y, Shimokawa H, et al. Testing a neural coding hypothesis using surrogate data. *J Neurosci Methods* 2008;172(2):312-22. doi: 10.1016/j.jneumeth.2008.05.004 [published Online First: 2008/06/21]
74. Goldstein A, Kapelner A, Bleich J, et al. Peeking Inside the Black Box: Visualizing Statistical Learning with PLots of Individual Conditional Expectation. *Journal of Computational and Graphical Statistics* 2015;24(1):44-65.
75. Vollert J, Maier C, Attal N, et al. Stratifying patients with peripheral neuropathic pain based on sensory profiles: algorithm and sample size recommendations. *Pain* 2017;158(8):1446-55. doi: 10.1097/j.pain.0000000000000935 [published Online First: 2017/06/09]
76. Blankenburg M, Boekens H, Hechler T, et al. Reference values for quantitative sensory testing in children and adolescents: developmental and gender differences of somatosensory perception. *Pain* 2010;149(1):76-88. doi: 10.1016/j.pain.2010.01.011
77. Hastie T, Tibshirani R. Generalized Additive Models. *Statistical Sciences* 1986;1:297-318.
78. Riley RD, Hayden JA, Steyerberg EW, et al. Prognosis Research Strategy (PROGRESS) 2: prognostic factor research. *PLoS Med* 2013;10(2):e1001380. doi: 10.1371/journal.pmed.1001380 [published Online First: 2013/02/09]
79. Freynhagen R, Baron R, Gockel U, et al. painDETECT: a new screening questionnaire to identify neuropathic components in patients with back pain. *Curr Med Res Opin* 2006;22(10):1911-20. doi: 10.1185/030079906X132488
80. Bouhassira D, Attal N, Fermanian J, et al. Development and validation of the Neuropathic Pain Symptom Inventory. *Pain* 2004;108(3):248-57. doi: 10.1016/j.pain.2003.12.024 [published Online First: 2004/03/20]
81. Craig CL, Marshall AL, Sjostrom M, et al. International physical activity questionnaire: 12-country reliability and validity. *Med Sci Sports Exerc* 2003;35(8):1381-95. doi: 10.1249/01.MSS.0000078924.61453.FB
82. Basu S, Poole J. The Brief Illness Perception Questionnaire. *Occup Med (Lond)* 2016;66(5):419-20. doi: 10.1093/occmed/kqv203 [published Online First: 2016/06/19]

For peer review only

Figure legends:

Figure 1: Study flow diagram

MRN: magnetic resonance neurography; SBI: sciatica bothersomeness index; NPRS: numerical pain rating scale.

For peer review only

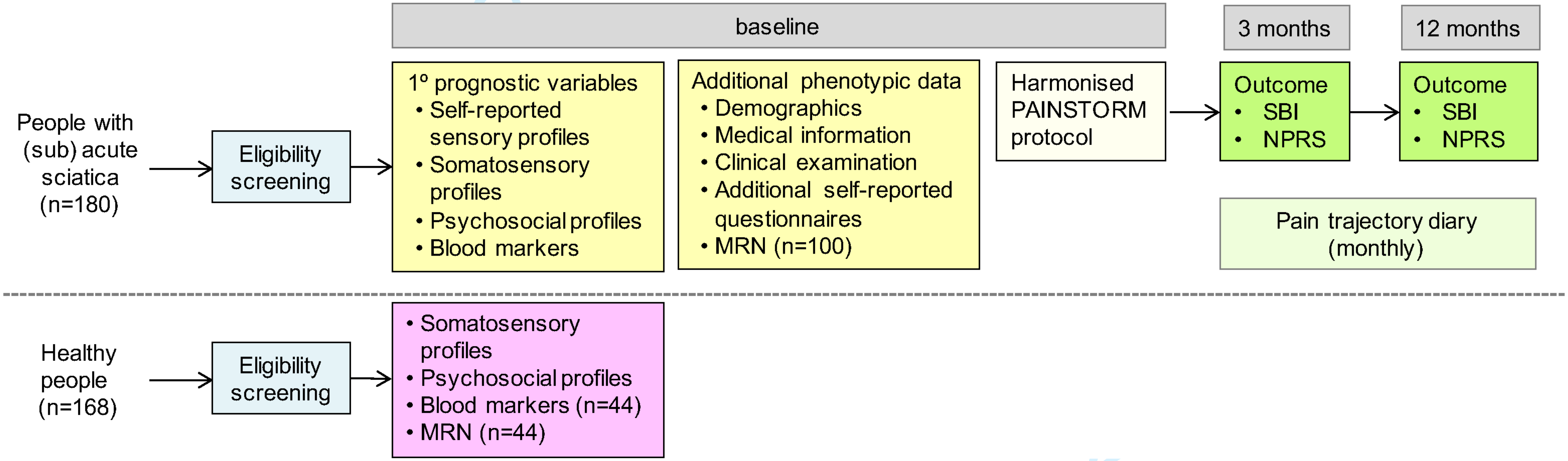
baseline
3 months
12 months
People with (sub) acute sciatica (n=180)
Eligibility screening
1° prognostic variables
• Self-reported sensory profiles
• Somatosensory profiles
• Psychosocial profiles
• Blood markers
Additional phenotypic data
• Demographics
• Medical information
• Clinical examination
• Additional self-reported questionnaires
• MRN (n=100)
Harmonised PAINSTORM protocol
Outcome
• SBI
• NPRS
Outcome
• SBI
• NPRS
Pain trajectory diary (monthly)
Healthy people (n=168)
Eligibility screening
• Somatosensory profiles
• Psychosocial profiles
• Blood markers (n=44)
• MRN (n=44)

Appendix 1: Pain trajectory Diary

ID______________
Date______________

Pain Trajectory Diary - FORECAST

Month:

Thank you for your continuing support of the FORECAST study. Please let us know below about your sciatica pain in the past 2 weeks.

	No pain 0	1	2	3	4	5	6	7	8	9	Worst pain imaginable 10
Sciatica leg pain											
**In the last two weeks, at its worst, how intense was your sciatica leg pain?**											
**In the last two weeks, at its least, how intense was your sciatica leg pain?**											
**In the last two weeks, on average, how intense was your sciatica leg pain?**											
Low back pain											
**In the last two weeks, on average, how intense was your back pain?**											

For peer review only

Appendix 2: Sciatica Perception Questionnaire

Your views about your sciatica (SPQ)

We are interested in your own personal views of how you currently see your sciatica.

Please indicate how much you agree or disagree with the following statements about your sciatica by ticking the appropriate box.

	Strongly disagree	Disagree	Neither agree nor disagree	Agree	Strongly agree
I expect that I am going into old age with my sciatica					
I feel that my sciatica will last for a long time					
My sciatica is likely to be permanent rather than temporary					
I expect that the effect of my sciatica on day-to-day life will worsen over time					
My sciatica comes and goes					
I do not know how my sciatica will change in the future					
My sciatica is a burden to others					
My sciatica can put me in awkward and embarrassing situations					
I have the personal strength to manage my sciatica					
I avoid specific positions and/or movements due to fear of causing pain					
I avoid specific positions and/or movements due to fear of causing damage					
There is little that I can do to improve my sciatica myself					
There is something seriously wrong with my back/leg					
The cause of my sciatica has not been investigated properly					
I am concerned about possible adverse long term consequences of the treatment for my sciatica					
There is nothing that can help my sciatica					
I do not understand what is wrong with my back/leg					
My current treatment does not make sense to me					
I worry that I am not getting the right treatment for my sciatica					
I don’t know what activities I can safely do with my sciatica					
It is so unfair that I have sciatica					

For peer review only

Appendix 3: Demographic and Medical History Data

Demographics	FORECAST substudy BASELINE	FORECAST substudy FOLLOW UP	Healthy Volunteers BASELINE	PAINSTORM DATASET EXTENDED
Age (yrs)	X		X	X
Sex	X		X	X
Years in education	X		X	X
Working status*	X		X	X
Household income**	X		X	X
Ethnicity	X		X	X
**Medical history**				
History of sciatica (date of first episode, number of previous episodes)	X			
Duration of current sciatica episode (days)	X			
Is the leg pain worse than the back pain?	X			
Affected leg (left/right/both)	X			
Family history of chronic pain	X		X	
Details of other medical diagnoses	X		X	
Cauda equina screening questions	X			
Types of treatments received for sciatica to date	X	X		
Types of tests/ investigations undertaken for sciatica to date	X	X		
Relevant previous and current medication, including whether or not they are taken for sciatica	X	X	X	
Medications: efficacy, adherence	X	X	X	X
Tobacco and Alcohol intake	X		X	X

* Working status:

- ☐ In paid employment or self-employed
- ☐ Retired
- ☐ Looking after home and/or family
- ☐ Unable to work because of sickness or disability
- ☐ Unemployed
- ☐ Doing unpaid or voluntary work
- ☐ Full or part-time student
- ☐ None of the above
- ☐ Prefer not to answer

** Which of the descriptions below comes closest to how you feel about your household's income nowadays?

For peer review only

- ☐ Living comfortably on present income
- ☐ Coping on present income
- ☐ Finding it difficult on present income
- ☐ Finding it very difficult on present income
- ☐ Do not wish to answer
- ☐ Don't know

For peer review only

Appendix 4: Clinical Examination

**Clinical Examination**<br>**(Identical for people with sciatica and healthy volunteers apart from assessments indicated with *which are performed only on people with sciatica)**	
Height (cm)	
Weight (kg)	
Waist circumference (cm)	
Hip circumference (cm)	
Myelopathy screening cluster	Tandem gait, inverted supinator sign, Hoffman's test, Babinski reflex
Neural mechanosensitivity*	straight leg raise, slump, femoral slump (where clinical picture indicates). Rated as negative or positive (at least partial symptom reproduction plus structural differentiation changes symptoms).
Lumbar spine active range of motion	flexion, extension, bilateral side flexion.<br>Range recorded as full or restricted.<br>Symptom provocation recorded as: none, leg, back, leg + back.
Palpation of lumbar spine*	Passive accessory intervertebral mobilisations (PAIVMS) over spinous processes L1-L5 centrally (to end of resistance if required).<br>Symptom provocation recorded as none, leg, back, leg + back

For peer review only

Appendix 5: MRI protocols

The MRN protocol includes multi-shell Diffusion Tensor Imaging (DTI) scans, high resolution coronal T1 and T2 weighted imaging, and T2 mapping scan, respectively.

- Multi-shell DTI consist of three coronal scans with three shots RESOLVE readout [56], TR/TE1/TE2=3430/46/81 ms, FOV = 256x256 mm^2, 2 mm isotropic spatial resolution, 26 slices, GRAPPA factor=2, and BW=1302Hz/Px. The first scan is acquired with b = 0 s/mm^2 and Left-Right (LR) phase encoding (PE) direction for the correction of susceptibility induced distortions. The Acquisition Time (TA) is 32 s. Each of the two other scans consist of 32 diffusion directions acquired with PE in RL direction and TA = 8min. The b-values are 700 s/mm2 and 1500 s/mm2, respectively.
- High resolution coronal T1 weighted images are acquired using a Turbo Spin Echo (TSE) sequence, TR/TE = 1050/11ms, FOV = 256x256 mm^2, 1 mm isotropic spatial resolution, 50 slices, 4 averages, GRAPPA factor = 2, Turbo Factor = 3, and TA = 6 min.
- High resolution coronal T2 weighted images are acquired using a TSE sequence, TR/TE = 4700/61ms, FOV = 256x256 mm^2, 1 mm isotropic spatial resolution, 50 slices, 4 averages, GRAPPA factor = 2, Turbo Factor = 15, with fat Saturation and TA = 9 min.
- Coronal multi-echo images are used to fit T2 maps. The acquisition parameters are: TR = 5700ms, TE = 13.8/27.6/41.4/55.2/69/82.8/96.6/110.4 ms, FOV = 256x256 mm^2, 1.3 isotropic spatial resolution, 40 slices, GRAPPA factor = 2, with fat saturation and TA = 9.5 min.

For peer review only
