# Supplementary material for: Factors predicting the transition from acute to persistent pain in people with ‘sciatica’: the FORECAST longitudinal prognostic factor cohort study protocol
Source: BMJ Open. 2023 Apr 5;13(4):e072832. doi: 10.1136/bmjopen-2023-072832 (PMC10111910; doi:10.1136/bmjopen-2023-072832)
Supplement: Reviewer comments [file bmjopen-2023-072832.reviewer_comments.pdf]

PEER REVIEW HISTORY

BMJ Open publishes all reviews undertaken for accepted manuscripts. Reviewers are asked to complete a checklist review form (**http://bmjopen.bmj.com/site/about/resources/checklist.pdf**) and are provided with free text boxes to elaborate on their assessment. These free text comments are reproduced below.

ARTICLE DETAILS

**TITLE (PROVISIONAL)**	Factors predicting the transition from acute to persistent pain in people with 'sciatica'-the FORECAST longitudinal prognostic factor cohort study protocol
**AUTHORS**	Schmid, Annina B.; Ridgway, Lucy; Hailey, Louise; Tachrount, Mohamed; Probert, Fay; Martin, Kathryn; Scott, Whitney; Crombez, Geert; Price, Christine; Robertson, Claire; Koushesh, Soraya; Ather, Sarim; Tampin, Brigitte; Barbero, Marco; Nanz, Daniel; Clare, Stuart; fairbank, jeremy; Baskozos, Georgios

This article was not externally reviewed at BMJ Open. Protocols that have been independently assessed prior to submission to BMJ Open are usually fast-tracked to publication on the grounds that further substantial changes will not be possible. This independent assessment will usually be external, independent review for both a substantial grant award from a non-commercial or government funder and ethics approval.
